# Shrouded in history: Unveiling the ways of life of an early Muslim population in Santarém, Portugal (8^th^– 10^th^ century AD)

**DOI:** 10.1371/journal.pone.0299958

**Published:** 2024-03-06

**Authors:** Rebecca Anne MacRoberts, Marco Liberato, Xavier Roca-Rada, Maria João Valente, Claudia Relvado, Teresa Matos Fernandes, Cristina Barrocas Dias, Bastien Llamas, Hermínia Vasconcelos Vilar, Bernd R. Schöne, Sara Ribeiro, José Francisco Santos, João C. Teixeira, Anne-France Maurer

**Affiliations:** 1 HERCULES Laboratory and IN2PAST, University of Évora, Évora, Portugal; 2 Centro de Estudos de Arqueologia, Artes e Ciências do Património (CEAACP), Universidade de Coimbra, Coimbra, Portugal; 3 Australian Centre for Ancient DNA, School of Biological Sciences, University of Adelaide, Adelaide, Australia; 4 Faculdade de Letras, University of Coimbra, Coimbra, Portugal; 5 Faculdade de Ciências Humanas e Sociais (FCHS), Universidade do Algarve, Faro, Portugal; 6 Interdisciplinary Center for Archaeology and Evolution of Human Behaviour (ICArEHB), University of Algarve, Faro, Portugal; 7 School of Technology Sciences, Department of Biology, University of Évora, Évora, Portugal; 8 Research Centre for Anthropology and Health (CIAS), University of Coimbra, Coimbra, Portugal; 9 School of Technology Sciences, Department of Chemistry and Biochemistry, University of Évora, Évora, Portugal; 10 Escola de Ciências Sociais—CIDEHUS, University of Évora, Évora, Portugal; 11 Institute of Geosciences, University of Mainz, Mainz, Germany; 12 Geobiotec, Department of Geosciences, University of Aveiro, Aveiro, Portugal; 13 Centre for Interdisciplinary Studies (CEIS20), University of Coimbra, Coimbra, Portugal; 14 Evolution of Cultural Diversity Initiative, The Australian National University, Canberra, Australia; Vilnius University: Vilniaus Universitetas, LITHUANIA

## Abstract

In around 716 AD, the city of Santarém, Portugal, was conquered by the Berber and Arab armies that swept the Iberian Peninsula and went on to rule the region until the 12^th^ century. Archaeological excavations in 2007/08 discovered an Islamic necropolis (Avenida 5 de Outubro #2–8) that appears to contain the remains of an early Muslim population in Santarém (8^th^– 10^th^ century). In this study, skeletal material from 58 adult individuals was analysed for stable carbon (*δ*^13^C_col_; *δ*^13^C_ap_), nitrogen (*δ*^15^N) and sulphur (*δ*^34^S) isotope ratios in bones, and stable oxygen (*δ*^18^O), carbon (*δ*^13^C_en_) and radiogenic strontium (^87^Sr/^86^Sr) isotopes in tooth enamel. The results of this study revealed a dietary pattern of predominantly C_3_-plant and domestic C_3_-fed herbivore consumption during adulthood (*δ*^13^C_col_ and *δ*^15^N, respectively) but a higher proportion of C_4_-plant input during childhood (*δ*^13^C_en_) for some individuals—interpreted as possible childhood consumption of millet porridge, a common practice in North Africa—in those with unorthodox burial types (Groups 1 and 2) that was not practiced in the individuals with canonical burials (Group 3). In this first mobility study of a medieval Muslim population in Portugal, *δ*^18^O_DW_ values revealed greater heterogeneity in Groups 1 and 2, consistent with diverse origins, some in more humid regions than Santarém when compared to regional precipitation *δ*^18^O data, contrasting the more homogenous Group 3, consistent with the local precipitation *δ*^18^O range. Ancient DNA analysis conducted on three individuals revealed maternal (mtDNA) and paternal (Y-chromosome) lineages compatible with a North African origin for (at least) some of the individuals. Additionally, mobility of females in this population was higher than males, potentially resulting from a patrilocal social system, practiced in Berber and Arab communities. These results serve to offer a more detailed insight into the ancestry and cultural practices of early Muslim populations in Iberia.

## 1.) Introduction

Relatively little is known about the lives of people living on the Iberian Peninsula during the eight centuries of Islamic rule. The chronicles tell great tales of military leaders and the key events that led to the conquering of various regions of Spain and Portugal, from varying perspectives, written in the decades and centuries following the conquest [1], but the day-to-day dietary or religious practices of people in various social strata are not readily visible in historical records. While historical sources can provide information about the types of foods that were available to people in the Middle Ages, and the rules that may have governed their consumption, they are not necessarily informative about the dietary habits of people [2, 3]. Archaeological evidence left behind by these societies has the potential to offer more insight into their material lives, the products that they used and traded, and how they made them, but this can rarely provide information on individual behavior or subgroups within a population. In contexts where archaeological burials are preserved, there exists a greater potential for understanding behaviour within past societies on a smaller, but more detailed scale, that of the individual. One aspect of this is how the individuals are buried, as their position or orientation within the grave could be indicative of religious burial rites, as well as what objects or materials might be present or absent in the burials, as is the case with Islamic necropolises. Another aspect is the skeletons themselves, from an anthropological point of view, biological characteristics can provide information on the age at death and biological sex of the individual as well as their general health and pathological conditions. In recent years, ancient DNA retrieved from skeletal remains has helped providing further biological information on the lives of ancient people, in particular by uncovering their population history and ancestral origin.

On a chemical level, the elements in the bones and teeth of these individuals can provide important information on their dietary and mobility patterns. Stable isotope analysis of carbon (*δ*^13^C) and nitrogen (*δ*^15^N) has become an increasingly common practice in archaeological studies of the diets of humans and animals living in the past [4–7] and more recently there is an expanding corpus of isotope studies focused on medieval Iberia although, to date, many more have been focused on medieval Spain [8–18] than on Portugal [19–22]. Published data on the mobility of medieval Iberian populations is even more scarce than dietary research, or indeed scarcer than mobility studies for other time periods in Iberia. To date, ^87^Sr/^86^Sr ratios have been used as a mobility indicator for early medieval Spanish populations in Álava [23] and Burgos [24], while the combination of ^87^Sr/^86^Sr ratios and *δ*^18^O values has been applied to understand mobility of medieval Islamic populations in Pamplona [25] and Tauste [15] as well as a transitional population in Biscay [26] in Spain. The mobility of medieval Muslim populations in Spain has also been addressed using *δ*^18^O, *δ*^13^C [18] and *δ*^34^S [27] analysis of archaeological skeletal materials in Ibiza. In Portugal, ^87^Sr/^86^Sr and *δ*^18^O have been used to interpret the mobility of a medieval Christian military order in Évora [22] but no multi-isotopic mobility studies have previously been published for a medieval Muslim population, despite the good preservation of archaeological skeletal material and their long period of occupation in the territory.

The use of biogeochemical information, stored in archaeological skeletons, to interpret aspects of past human lives such as dietary choices and mobility contributes to a more detailed and nuanced understanding of how societies interacted with their environments and with each other in the past. This is of particular relevance in Portugal, where multi-isotopic studies on archaeological populations are still scant, and even more so for the early centuries of Islamic occupation, a time of significant cultural and political change that is nevertheless quite poorly documented. In this study, biogeochemical data obtained from archaeological skeletons from the site of Avenida 5 de Outubro #2–8, was combined with archaeological, anthropological, genetic and historical information in order to better understand how this early (ca. 8^th^-10^th^ century) Muslim population lived and thrived in the newly conquered city of Santarém, Portugal.

## 2.) Isotopes, diet and mobility

The main premise upon which isotope dietary studies are based, is that dietary input directly influences the *δ*^13^C and *δ*^15^N values found within the tissues of an individual [28]. The photosynthetic pathways of C_4_ plants (e.g. maize, sorghum, millet, sugar cane etc.) differ to C_3_ plants (e.g. wheat, barley, rice, shrubs, trees, etc.) resulting in different discriminations of carbon-13 relative to carbon-12 in the respective plant tissues [29, 30]. These *δ*^13^C values are also reflected in the tissues of consumers of the plants–stable carbon isotope ratios in bone collagen (*δ*^13^C_col_) mostly come from the protein inputs of the diet while those in bone apatite (*δ*^13^C_ap_) and tooth enamel (*δ*^13^C_en_) are contributed by whole diet (carbohydrates, lipids and proteins) [31, 32]. The analysis of these components can reveal important information about the averaged dietary input in the last years of life (in bones) or in early childhood over the time interval of tooth crown formation [28]. *δ*^15^N values in consumers are typically more positive than those of their food sources as dietary ^15^N is preferentially incorporated over ^14^N [33]. Thus, *δ*^15^N values in bone collagen can indicate trophic level within food webs, and in archaeological contexts the most accurate assessments of dietary relationships can be made when human and faunal bones from the same archaeological context are analysed [34], a practice that also helps to overcome environmental variability in isotopic values [35]. The combination of *δ*^13^C and *δ*^15^N values can also be useful for distinguishing between terrestrial and marine protein sources [36–38].

In addition to providing information about dietary habits in the past, isotopes in skeletal tissues can also be useful for better understanding the movement of populations and individual mobility, using regional atmospheric and geological ‘signatures’ for comparison [39]. Tooth enamel is much more resistant to diagenetic change than bone apatite due to its crystalline structure [40], and once it is formed, it is chemically inert, so it has the potential to retain the oxygen and strontium isotope values incorporated during an individual’s childhood. Oxygen isotopes in human or animal tissues reflect the isotope values of body water, which in turn is determined by the oxygen isotope (*δ*^18^O) values of the water ingested (in drinking water and food) during the tissue formation [41]. Ingested water typically comes from local groundwater and surface water in archaeological populations, and the *δ*^18^O values of groundwater generally reflect local meteoric precipitation values, which vary spatially and temporally [42]. Factors such as latitude, altitude, surface temperature, distance to sea and the amount of precipitation can all influence rainfall *δ*^18^O values, meaning that regional variability can be used as a basis against which to compare *δ*^18^O values in human tissues. The oxygen isotope values in tooth enamel carbonate and phosphate (*δ*^18^O_c_ and *δ*^18^O_p_) require a conversion to drinking water (*δ*^18^O_DW_) values to account for metabolic fractionation before a comparison can be made to regional *δ*^18^O values [43, 44], although it should be taken into consideration that these conversions can introduce additional uncertainty [45]. In recent years, isoscapes developed from archaeological data have proven useful in mobility studies, as they reduce the uncertainty involved in the conversion of archaeological tooth enamel *δ*^18^O_DW_ values, which is necessary for a comparison with regional precipitation values in order to identify “non-locals” [46, 47]. The current lack of published *δ*^18^O_p_ data for contemporaneous medieval populations in Iberia makes it difficult to rely on average population values to predict ‘local’ values for Santarém, as has been done elsewhere in Europe for archaeological populations [48–50]. Therefore modern precipitation *δ*^18^O values are a useful source for comparison at specific sites, if data is available from GNIP, the Global Network of Isotopes in Precipitation, for example [51] or in the form of modelled precipitation isoscapes [52–54]. It should be noted that in some regions, such as the Iberian Peninsula, regional variability in *δ*^18^O values is relatively low, therefore it is most useful when combined with other isotope systems for mobility studies.

Radiogenic strontium isotope ratios (^87^Sr/^86^Sr) are the most commonly used mobility indicator in archaeological studies. Strontium can substitute for calcium in hydroxyapatite during tooth formation due to the chemically similar properties of these elements, and since strontium reflects geological regional variability and does not fractionate in biological processes, tooth enamel ^87^Sr/^86^Sr values can be compared to regional ones, to identify individuals who migrated to an area after childhood [55, 56]. ^87^Sr/^86^Sr is a function of the relative abundances of rubidium and strontium, and indicates the age and type of rocks because ^87^Sr is formed by the β-decay of ^87^Rb over time, thus older rocks will have a higher ^87^Sr content i.e. a higher ^87^Sr/^86^Sr ratio while more recently formed rocks will have lower ^87^Sr/^86^Sr ratios [39]. Soil ^87^Sr/^86^Sr ratios broadly reflect those of the weathered bedrock, but minerals in the bedrock weather at different rates and groundwater introduces additional variability, so the geological ^87^Sr/^86^Sr ratios will not necessarily be the same as the bioavailable ^87^Sr/^86^Sr that is absorbed by the plants growing in the soil and subsequently incorporated into the tissues of the consumers of those plants [57]. To overcome this problem, ^87^Sr/^86^Sr is measured in various sample types (e.g. soil, water, plants) to build a regional database or isoscape, in order to estimate the expected local ^87^Sr/^86^Sr range for an archaeological site. Caution should be taken to avoid modern anthropogenic contamination in samples [58, 59], but the generation of isoscapes is nevertheless an invaluable resource for the interpretation of mobility patterns in archaeological individuals and/or populations [47, 60, 61].

Stable sulphur isotopes (*δ*^34^S) in bone collagen are a product of the isotopic composition of food consumed, and the sulphur in plants originates mainly from sulphate in the soil and the atmosphere [62]. Sea spray and aerosols can transfer high marine *δ*^34^S values (>15‰) to terrestrial environments, where they are incorporated into plants growing in coastal areas [63]. The uneven weathering of rock minerals can also result in variability of geological sulphur content, with the contribution of bedrock as a sulphur source to ecosystems appearing to be higher in regions where the bedrock is rich in sulphur (e.g. evaporites) [47, 63]. The amount of fractionation between the environmental and assimilated sulphur in plants is highly variable as a result of the mixing of these isotopically distinct sources [64], but once the organic bound sulphur enters the food web (as amino acids within proteins) there is only a small amount of fractionation between food sources and consumers [65]. While marine ecosystems show relatively little *δ*^34^S variability, freshwater ecosystems show large ranges of *δ*^34^S depending on the geological contributions and source of water sulphates [62]. In archaeological populations, food is generally expected to be sourced relatively locally, thus *δ*^34^S values in skeletal tissues should reflect averaged contributions of local ecosystems and may be useful as an additional indicator of mobility [47].

## 3.) Context

### 3.1.) Regional context

#### 3.1.1) Geographic and geological context

**Fig 1:**
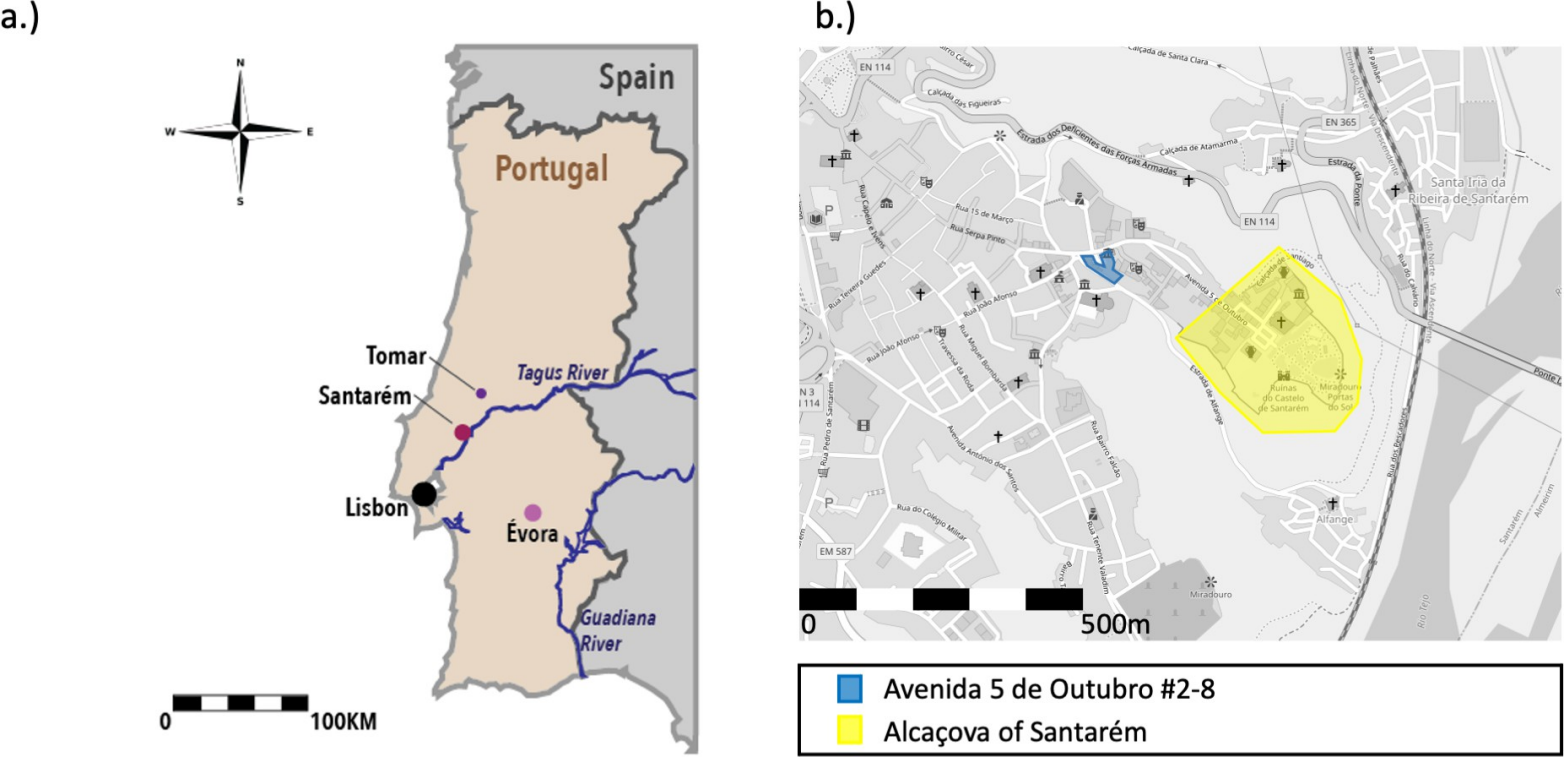
Map of a.) Location of Santarém and b.) Excavated area of Avenida 5 de Outubro. Map in Fig 1A). drawn by MacRoberts RA and adapted from Wikimedia Commons (https://commons.wikimedia.org/wiki/File:Portugal-CIA_WFB_Map.png), CC public domain. Map in Fig 1B.) taken from openstreetmap.org licensed under the Open Data Commons Open Database Licence (ODbL).

**Fig 2:**
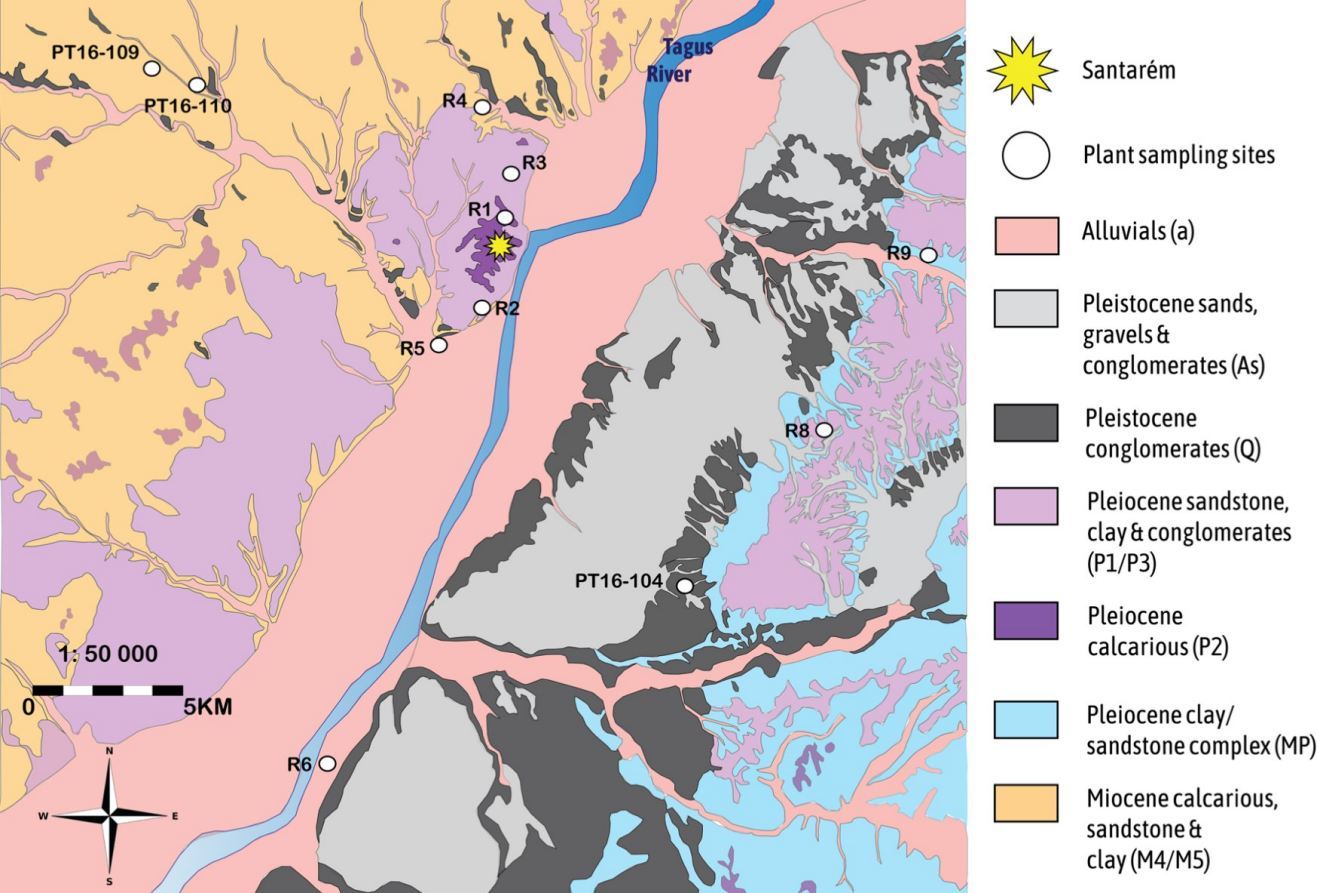
Geological map of Santarém and sampled sites for bioavailable ^87^Sr/^86^Sr baseline. Map drawn by MacRoberts RA and adapted from the LNEG (Laboratório Nacional de Energia e Geologia) 1: 50 000 geological raster maps “31-A” and “31-C”. (https://geoportal.lneg.pt/pt/dados_abertos/cartografia_geologica/cgp50k/) [67]. Both geological raster maps 31-A and 31-C are published under CC By 4.0. (31-A: https://sig.lneg.pt/metadados/catalog/search/resource/details.page?uuid=831fb1df-e743-4e23-97d0-91af58d46f02; 31-C: https://sig.lneg.pt/metadados/catalog/search/resource/details.page?uuid=20486b7f-20be-4506-aa88-5caacc5d6946).

The city of Santarém is located in the southwest of the Iberian Peninsula, in the Tagus River Valley (Fig 1A). Its proximity to the river was strategically advantageous for trade in the past while the fertile alluvial plain would have been good for food production. The hilltop position of the settlement, elevated approximately 90m above the river, made it easier to defend during its turbulent history [66]. Santarém is situated on the Middle sector of the Lower Tagus Cenozoic Basin and is covered by Miocene and Pleiocene sedimentary deposits followed by Pleistocene fluvial terraces (Fig 2). The Pleiocene plateau on which the city rests contains units of limestone, clay and marl (P2) and layered sandstone and claystone (P1) [67]. Within a few kilometres, there are Miocene outcrops of limestones, sandstones and clays (MP, M4) and modern deposits (a) on the alluvial plain west of the river and Pleistocene alluvial terraces (Q3) [67, 68]. Plant samples (collected and measured by [61]) from within 15Km of Santarém yielded ^87^Sr/^86^Sr ratios of 0.712 and 0.710 where the geological substrate is Miocene calcareous material and sandstone, and a ^87^Sr/^86^Sr ratio of 0.715 where the substrate is Pleistocene gravels, sands and limestone [61].

#### 3.1.2) Environmental background for *δ*^18^O and *δ*^34^S

Based on the extrapolated mean annual precipitation data provided on the “WaterIsotopes” database [53, 54, 69], modern regional values for Portugal range between -5.5‰ and -3.5‰ over most of the territory and between -7.5‰ and -5.5‰ in the less arid regions to the north of the Tagus River. While there is no GNIP (Global Network of Isotopes in Precipitation) data for Santarém itself, the closest stations with recorded data are Portalegre (100km due E) with an annual weighted mean *δ*^18^O value of -5.91 ± 0.63‰ (1988–2004) and Lisbon (70km to the SSW) with an annual weighted mean *δ*^18^O value of -4.55 ± 0.57‰ (2014–2017) [51]. The precipitation *δ*^18^O isoscape modeled by Hatvani *et al*. [52], based on GNIP data recorded in the Iberian Peninsula from 2004–2006, also predicts an average range of ~-5‰ to -4‰ for Santarém. Precipitation *δ*^18^O values lower than ~-6‰ may occur in the winter in Santarém when rainfall is higher but not typically over long term averages, while the mean annual *δ*^18^O values can range from -4.2‰ to -11.1‰ over the Maghreb region of northern Africa with the most negative values being found over the Atlas Mountains [53, 54, 69, 70]. It is important to consider that tooth enamel is formed over long time periods, so annual or long terms means are more comparable than seasonal data for bulk tooth samples [71].

Regarding sulphur, the *δ*^34^S isoscape developed by Bataille *et al*. [47], based on post-Mesolithic human and animal teeth, predicts a range of ~12‰ to 16‰ for central and southern Portugal although only two sets of published data from Portugal were available to be included in this model. These datasets included human and fauna *δ*^34^S from the sites of Tomar [19] and Évora [22], both of which had higher faunal *δ*^34^S values than the human populations, and were unexpectedly high for two inland sites. In the case of Tomar, the high proportion of evaporitic bedrock [72] was thought to contribute to the high *δ*^34^S values of the fauna, while a large proportion of freshwater fish intake influenced the lower human *δ*^34^S values [19]. In Évora, high influx of Saharan aeolian deposit bringing marine sulphates were thought to elevate the environmental *δ*^34^S, indicated by the faunal values and the human population was largely expected to be non-local [22]. This means that the *δ*^34^S model developed by Bataille *et al*. [47] included, among other variables (geology, environment), possibly ‘atypical’ cases for Portugal and should be used with caution for this region for the time being. The nearest coastline to Santarém is ~50 km away, and the likelihood of seaspray effect, a factor which commonly results in higher regional *δ*^34^S values [62], is not expected to be high. It is worth considering that the climatic periods of the Dark Ages (DA: 500–900) and the Medieval Climate Anomaly (MCA 900–1300 AD) brought an influx of persistent winds from northwest Africa and high Saharan aeolian input across the southern Iberian Peninsula, as evidenced by palaeoclimatic studies in the Algerian-Balearic Basin [73] and the Tagus River Basin [74, 75]. The highest Saharan aeolian input occurred at the end of the DA (650–900 AD) and during the Early MCA (900–1100 AD) which coincides with the likely time of deposition of the Islamic graves in this necropolis.

### 3.2.) Historical context

In 711 AD, the Islamic conquest of Iberia began, led by the General Tariq Ibn Ziyad. The Berber armies, under the rule of the Damascus-based Umayyad dynasty and united by their Islamic faith, crossed from Tangiers into Gibraltar. What may have begun as raids rapidly escalated into a full-on conquest of the peninsula [76, 77]. After defeating the Visigoth king, Roderic, the Muslim armies, reinforced by Arabs under the command of Musa ibn Nusayr, met little resistance and swiftly conquered Córdoba, Toledo (the Visigothic capital) and Mérida in 713 AD. By 716 AD, the cities of Évora, Santarém and Coimbra had fallen. Within just five years, Muslim forces had gained control of the entire Iberian Peninsula, with the exception of Asturias, a mountainous region in the northwest that remained under Christian control [78]. Favoring the drier climate south of the Tagus river, the Islamic dominion over the region would continue until the 12^th^ century in most of Iberia and as late as 1492 AD in Granada.

The newly settled population consisted of mainly North African Berbers with Arabs making up the higher social status [76]. The Umayyad dynasty was primarily concerned with military expansion and the conquering of territories rather than proselytisation. It did not favor the conversion of non-Muslims, and Christians and Jews living in “*Shantarîn*” and other cities would likely have been allowed to keep their religion and property with the protected legal status of “*dhimmi*”, on the condition that they paid a yearly tax or “*jizya*” [79]. In fact, conversion was at times actively discouraged by the Umayyads as it would impact state revenue, since Muslim landowners paid a lesser tax or “*ushr*” [78, 80]. In the mid 8^th^ century, following Berber Revolts and civil unrest, the Umayyad Caliphate was brought down by the Abbasids who shifted the main seat of control from Damascus to Baghdad [81]. Most of the Umayyad’s were slaughtered in the coup, with the exception of Abd al-Rahman, who fled to Spain and founded a new branch of the Umayyad Dynasty, the “Emirate of Córdoba” in 756 AD which continued to rule Iberia independently for a further three centuries [76]. In the 10^th^ century, control of al-Andalus was divided amongst the *taifa* kingdoms (the most important being Badajoz, Toledo, Zaragoza and Seville), followed by the respective rules of the Berber Almoravids and the Almohads [78, 80]. This fragmentation of Muslim control in the 11^th^ and 12^th^ centuries paved the way for the Christian conquest and eventual establishment of the Portuguese Kingdom. Santarém, an important cultural centre under Islamic rule, was captured by King Afonso Henriques and the Christian forces on 15 March 1147 [82].

During the period of Islamic rule, al-Andalus likely enjoyed trade and commercial relationships with other Islamic regions [80], facilitating a cultural expansion as much as a military one. Technological innovations brought by the Arabs to the Iberian Peninsula included new irrigation methods, experienced as they were with conserving and distributing water in the Middle East. With new irrigation techniques came the potential for growing crops that required an adequate water supply, hence the Muslims introduced cotton, rice, brinjal, artichoke, apricot, banana, date palm, spinach, coconut, orange and lemon, and this legacy is evident in some of the names of these crops in Portuguese and Spanish that derive from Arabic [80, 83]. Also, among these introduced crops, are two very important C_4_ plants: sorghum and sugarcane. Sorghum was cultivated in the Niger Delta and Nile Valley, spread to the Arabian Peninsula and Asia [84], and was later brought to Europe with the Islamic conquest. It is highly drought-tolerant and requires at least 35 cm of rain per year, slightly more than millet. It is still commonly eaten in Africa in the form of bread, boiled like rice or as a porridge. Sugarcane is a tropical grass that was cultivated in southeast Asia around 9000 BC, spread to India and then Persia along the Silk Road around 500 AD [85]. In the 7^th^ and 8^th^ centuries, Muslim traders brought sugar to North Africa and southern Iberia, with the most refined quality being produced in high quantities in Egypt. Its poor nutritional value, however, made it a delicacy but not a staple crop.

Millet, a hardy C_4_ plant originally cultivated in Africa (pearl millet and finger millet) and Asia (proso millet and foxtail millet), grows well in infertile, sandy soil and requires as little as 25 cm of rain per year. It was grown by Europeans by the third millennium BC but became widespread around 1000 BC [85] and thus is was the only prevalent C_4_ plant to be consumed in Europe before the Muslim conquest. In the Middle Ages, it was commonly consumed in porridge and flatbread, and is still a major food source in modern African and Asian populations.

Concerning medieval Islamic burials, tradition dictated that the head of the deceased was turned to the right and pointed towards the *Kaaba* in Mecca, as instructed by the *hadiths*, a direction known as the *qibla* [86]. However, it has been noted that the orientation of the *qibla* varied across regions and the way it was sought differed greatly [87]. No mathematical solution existed for identifying this direction prior to the 8^th^ century, and in some ancient Muslim traditions Jerusalem or Petra may have been chosen for the direction of prayer, rather than Mecca [87–89]. Although this may mean that many ancient Muslim burials may not have been in the ‘correct’ canonical position, the most important thing for the believer would be the intention of facing the *qibla*, even if it is not geographically correct in the end.

At a shorter timescale, it has been observed that variations in the orientation of graves, by as much as 35°, could have occurred as a result of seasonal differences in the angle of the sun because, in the absence of a permanent indicator, grave diggers could orient graves using the rising or setting sun [90].

### 3.3.) Archaeological context

#### 3.3.1.) Avenida 5 de outubro

**Fig 3:**
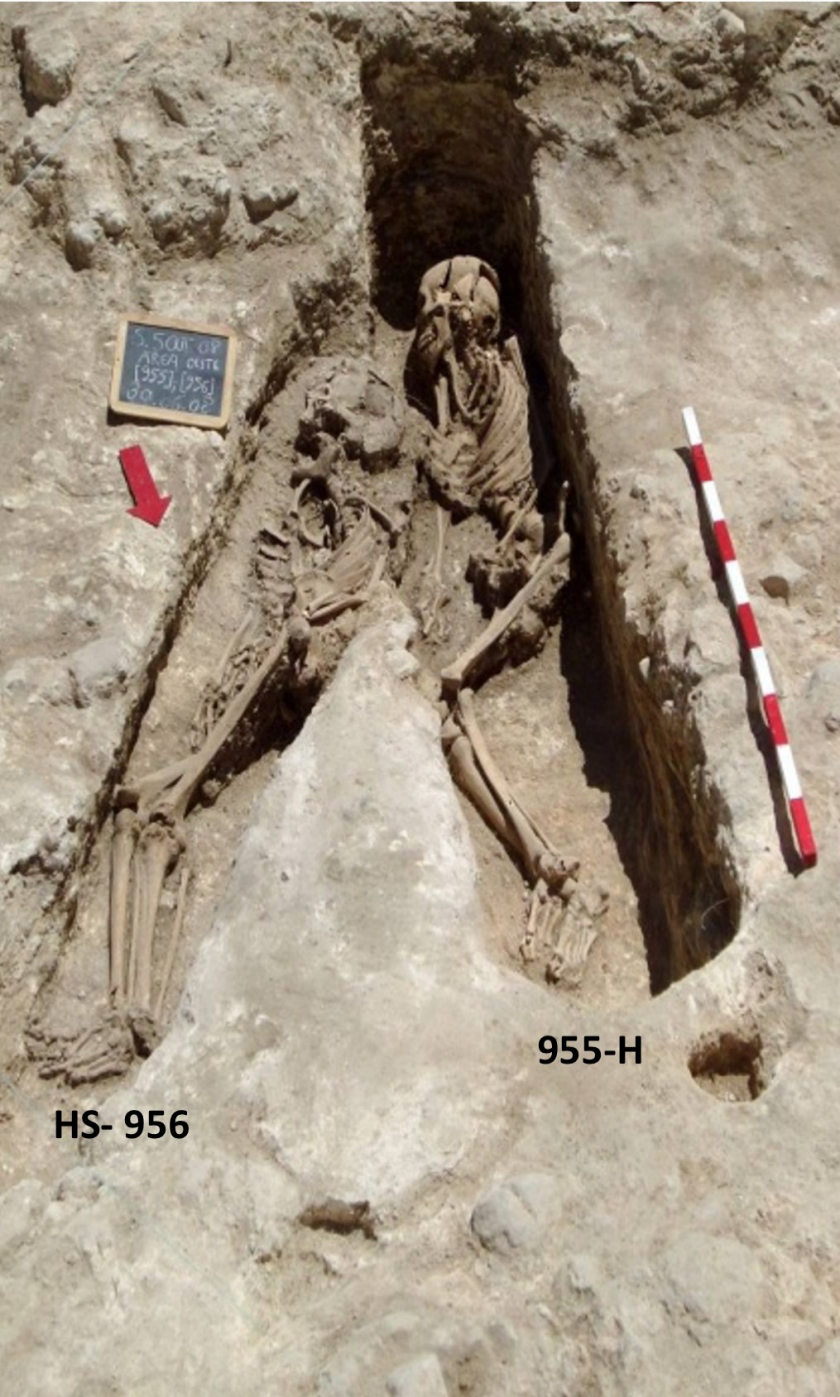
Image of intersecting graves 955 and 956 with differing orientations. Republished from [91] under a CC BY license, with permission from *Medievalista*, original copyright 2012.

During the 8^th^ & 9^th^ centuries, the inhabited area of Santarém would most likely have been concentrated in the *alcáçova* (castle), located on the highest and most central part of the hilltop settlement. A small neighborhood was located to the northeast and outside of the walled area (Fig 1b), but was later incorporated into the city as the population grew and the defensive line was extended North into the Marvila Hill by the 11^th^/12^th^ century [91]. The site of Avenida 5 de Outubro #2–8 (WGS84: 39.235627; -8.679350), in Alporão, Santarém, was excavated during 2007 and 2008, uncovering a necropolis that contained evidence of use during the Roman, Visigothic and Islamic Periods. 58 adult and 32 non-adult skeletons were recovered from the Islamic necropolis. The graves of interest to this study consisted of simple burial pits, with an absence of grave goods and the individuals placed on their right side, and thus appeared to be consistent with medieval Islamic burial rites whereby the dead are placed in a shroud, unaccompanied by ornamentation and laid on their sides in a simple grave [86]. Some of the graves in the northern part of the excavated area contained skeletons that were oriented S/N, a slight deviation from the orthodox position whereby they should be aligned to the *qibla*, while skeletons in graves in the southern and south-eastern area are all oriented SW/NE and canonically aligned to the *qibla* (head to the SW, body on the right side and face turned towards Mecca i.e. facing a south-eastern direction) [91] (Fig 3).

Originally interpreted as related to chronology, the misaligned (S/N-oriented) burials were thought to be a consequence of early conquest settlers (themselves probably newly-Islamised Berbers) converting existing Christian temples into mosques and incorrectly estimating the direction of the *qibla* [91]. In the late 10^th^ century, under the initiative of Caliph Hisham II, the *aljama* mosque was constructed in Santarém [92], at which point the *mihrāb* would have been corrected (a niche in the mosque wall that indicates the direction to pray) and it was initially supposed that burial orientations would have been standardised accordingly i.e. SW/NE-oriented graves in this necropolis [93]. A recent reappraisal of the stratigraphy data (exceeding 2500 units) has led to reconsideration of the chronological relationships and revealed that some of the misaligned burials might in fact be more recent than the canonical ones, such as the S/N-oriented grave of skeleton 955 which overlaps the canonical grave of skeleton 956 (Fig 3). An ongoing investigation by a separate research team at the University of Coimbra will provide radiocarbon dates adding further information on the chronology of the site.

Taking into account the medieval Islamic preference for burying the dead outside the cities of the living [86, 93], and archaeological evidence of the expansion of the city over time, Liberato and Santos (2017) [93] have hypothesised that the necropolis of Avenida 5 de Outubro was used between the 8^th^ and 10^th^ century by some of the earliest Muslim inhabitants of Santarém following the conquest (and the earliest to bury their dead in the necropolis). The presence of large negative structures, probably silos, dating to the first *taifas*/Almoravid period indicate that by the early 11^th^ century, this space was no longer primarily used as a necropolis [91]. By this point, another necropolis had been established further west on the plateau, a probable result of the growing population and extended defensive line of Santarém.

#### 3.3.2.) Villa rosa palace

Faunal specimens were recovered from medieval deposits in the 2008 excavations of Avenida 5 de Outubro #5–8 (Villa Rosa Palace) in Santarém (91,93). Samples came from silo infill (unit 1666), interpreted as a late Islamic context (after 10^th^ century) and from a pit (1598) typical of an Islamic/Christian transitional context, containing Islamic style ceramics and some post-conquest materials (e.g. 13^th^ century coins).

## 4.) Materials

### 4.1.) Human skeletons

Approval for collecting, sampling and destructive analysis, was granted by the Câmara Municipal of Santarém, Portugal, where the skeletal specimens under study are curated. According to Portuguese laws and regulations concerning archaeological human remains, no permits were required for the described study. Prior to selection for chemical analysis, the human skeletons from Avenida 5 de Outubro were subject to anthropological assessment of age at death and sex. Sex estimation was determined by evaluating both pelvic and cranial features, following methods established based on Portuguese reference collections [94] and recommended standards [95]. The age at death of adult skeletal remains examined in this research were established using degeneration of the pubic symphysis [96], degeneration of the auricular surface [97], and recommended standards [95]. The results of age at death estimations were grouped into three age categories: young adults ranging from 18 to 29 years, mature adults between 30 and 50 years, and older adults over 50 years of age.

Following this assessment, bones from 58 adult skeletons (25F, 20M, 13U) were selected for *δ*^13^C, *δ*^15^N and *δ*^34^S analysis. Long bones were preferentially selected because of their long turnover rates, incorporating dietary input over a period of more than a decade [98] and high volume of compact bone which facilitates good collagen preservation [99]. In the case of HS-811, no long bones were available and a rib was sampled instead, which may represent a different period of life prior to the death of the individual [98]. Wherever possible, broken bones were chosen for sampling in order to minimise damage to archaeological material. Of the bones selected for *δ*^13^C and *δ*^15^N analysis of the bone collagen, 45 were also sampled for *δ*^13^C analysis in the bone apatite, so that whole diet carbon contributions could be considered alongside the protein dietary inputs. Although 25 skeletons did not have teeth preserved, either due to ante-mortem loss or taphonomic processes, tooth enamel from 22 individuals was sampled in order to measure *δ*^18^O and ^87^Sr/^86^Sr for the reconstruction of mobility patterns, as well as *δ*^13^C in order to assess whole diet inputs during childhood. Loose molars were preferentially selected to avoid damaging well preserved jaws wherever possible. First molars (M1s) were preferred for their potential to yield birthplace ^87^Sr/^86^Sr signatures [100], but when they were not available, M2s or M3s were sampled. The enamel of tooth crown forms between the ages of birth—~2 y in the M1, ~18 mths—~5 y in the PM1, ~3 - ~6.5y in the PM2, ~3.5 - ~6.5y in the M2 and ~9.5 – 12y in the M3 [101]. In order to account for possible oxygen isotope enrichment (higher *δ*^18^O_DW_ values) in the first molar (M1) tooth enamel as a result of breastfeeding, which may occur as a result of fractionation in the body water of the mother [41], wherever possible a later formed tooth (M2/M3) was sampled in addition to the M1s. Of these 22, 9 individuals were selected for the analysis of two teeth each for *δ*^18^O and *δ*^13^C, with the aim of checking for differences in teeth formed earlier vs later (as per availability) that could be introduced by a breastfeeding signal, or by changes in diet or location during childhood. Six individuals were also selected for ancient DNA analysis, three of which had maxillofacial prognathism, broad nasal apertures, smooth inferior nasal margins, retracted chins and rectangular occipital orbits, features that are more prevalent in pre-modern populations with African genetic ancestry than Eurasians [102, 103], and thus their genetic lineage was of particular interest in this context. Ancient DNA molecules were extracted from the petrous bones of these six skeletons. All materials are listed in Table 1.

**Table 1:** Human skeletal material.

Sample	Bone	Tooth	Age	Sex	Grave/Skeleton Orientation	Group	Prognathism	DNA
HS-557			Mature Adult	M	S/N Oriented	1		
HS-561	Fibula		Adult	Maybe F	S/N Oriented	1		
HS-931		PM2 M3	Mature Adult	F	S/N Oriented	1	Y	Y
955-H	Humerus	M1	Young Adult	F	S/N Oriented	1	Y	FAILED
HS-1027	n/a	M3	Adult	F	S/N Oriented	1		
HS-1061	n/a	M2	Adult	Maybe F	S/N Oriented	1		
HS-1156	Femur	PM2 M2	Adult	M	S/N Oriented	1		
1442-F	Femur	M2 M3	Young Adult	F	S/N Oriented	1		
1647-F	Femur	M3	Mature Adult/Old	M	S/N Oriented	1		FAILED
HS-1865	Femur		Mature Adult	M	S/N Oriented	1		
HS-1979	Femur		Young Adult	M	S/N Oriented	1		
HS-1985	Humerus		Adult	F	S/N Oriented	1		
HS-2003	Femur	M2	Adult	F	S/N Oriented	1		
HS-2242	Femur	M2	Young Adult/Mature	F	S/N Oriented	1		
HS-2271	Femur		Adult	F	S/N Oriented	1		
HS-2273	Femur		Mature Adult	M	S/N Oriented	1		
HS-2316	Humerus		Mature Adult	M	S/NS Oriented	1		
2334-F	Femur	M1 M3	Young Adult/Mature	M	S/N Oriented	1		Y
HS-612	Femur	M2	Mature Adult	M	Maybe S/N Oriented	2		
639-F	Femur	M1	Mature Adult	F	Maybe S/N Oriented	2		
HS-761	Humerus		Adult	F	Maybe S/N Oriented	2		
HS-918	Femur		Mature Adult	F	Maybe S/N Oriented	2		
HS-921	Femur		Adult	M	Maybe S/N Oriented	2		
1092-F	Femur	M1	Adult	M	Maybe S/N Oriented	2		FAILED
HS-1251	Femur	M3	Adult	F	Maybe S/N Oriented	2		
HS-1491	Femur		Adult	F	Maybe S/N Oriented	2		
HS-1584		M1 M3	Young Adult	F	Maybe S/N Oriented	2	Y	Y
HS-1604	Tibia		Adult	U	Maybe S/N Oriented	2		
2075-F	Femur	M3	Mature Adult	F	Maybe S/N Oriented	2		
HS-2128	Femur	PM1 M3	Young Adult	F	Maybe S/N Oriented	2		
HS-2275	Femur		Mature Adult	M	Maybe S/N Oriented	2		
HS-559	Tibia	M1 M2	Mature Adult	F	SW/NE Oriented	3		
HS-811	Rib		Adult	U	SW/NE Oriented	3		
HS-819	Femur		Adult	M	SW/NE Oriented	3		
HS-867	Femur		Mature Adult	U	SW/NE Oriented	3		
HS-923	Femur		Adult	F	SW/NE Oriented	3		
HS-956	Femur	M2	Old Adult	F	SW/NE Oriented	3		
HS-958	Femur		Adult	M	SW/NE Oriented	3		
HS-1103	Humerus		Adult	U	SW/NE Oriented	3		
HS-1135	Femur		Mature Adult	U	SW/NE Oriented	3		
HS-1325	Femur		Adult	Maybe F	SW/NE Oriented	3		
HS-1480	Femur		Mature Adult	F	SW/NE Oriented	3		
HS-1619	Femur		Adult	Maybe M	SW/NE Oriented	3		
HS-1622	Femur		Adult	M	SW/NE Oriented	3		
1782-F	Femur		Young Adult	M	SW/NE Oriented	3		
HS-1794			Mature Adult	M	SW/NE Oriented	3		
1976-F	Femur	M1 M2	Adult	M	SW/NE Oriented	3		
HS-2079	Femur	M1 M3	Old Adult	F	SW/NE Oriented	3		
HS-2082	Femur		Adult	F	SW/NE Oriented	3		
HS-2170	Femur		Adult	M	SW/NE Oriented	3		
HS-2181	Femur		Adult	U	SW/NE Oriented	3		
HS-2189	Femur		Adult	Maybe F	SW/NE Oriented	3		
HS-2215	Femur		Mature Adult	Maybe M	SW/NE Oriented	3		
HS-2320	Humerus	M3	Mature Adult	M	SW/NE Oriented	3		
HS-2356	Femur		Mature/Old Adult	F	SW/NE Oriented	3		
HS-2358	Femur		Mature Adult	F	SW/NE Oriented	3		
HS-2361	Femur		Mature Adult	F	SW/NE Oriented	3		
HS-2423	Tibia		Adult	Maybe M	SW/NE Oriented	3		

Samples sorted according to the Grave Orientation (groups 1, 2 and 3), followed by sample number.

#### 4.1.1.) Grave orientation

**Fig 4:**
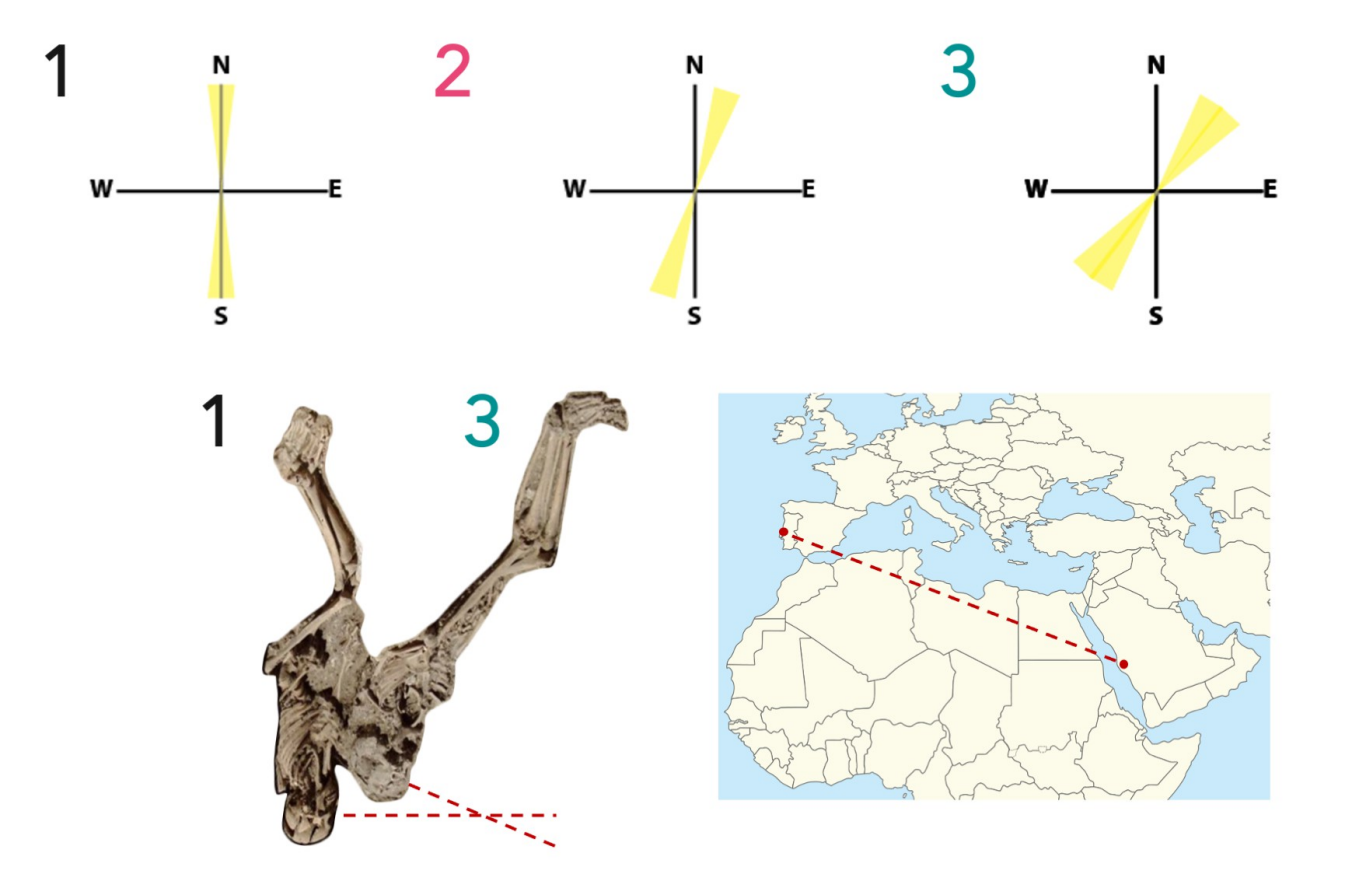
Grave orientation classification, diagram showing the direction that skeletons would face in right lateral position with faces turned towards the east or southeast, and the direction from Santarém towards Mecca. Drawn by MacRoberts RA. The map image inset was obtained from Wikimedia Commons (https://commons.wikimedia.org/wiki/File:North_Africa_location_map.svg), CC public domain. The skeletons were adapted from [91] with permission from Medievalista, original copyright 2012, republished under a CC BY license.

**Fig 5:**
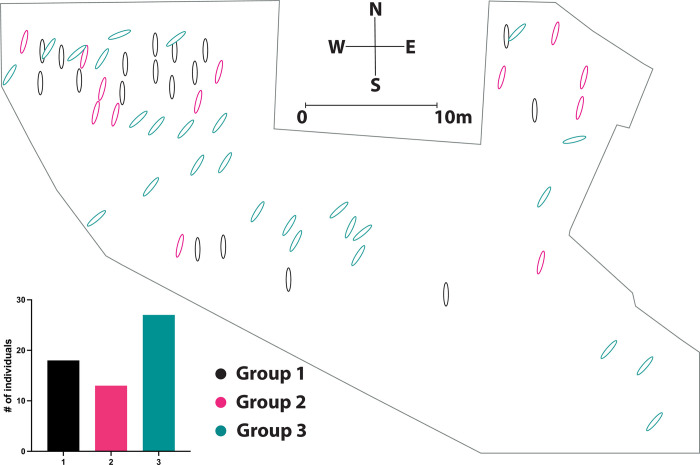
Map of Avenida 5 de Outubro necropolis showing position of graves and Bar graph showing # of individuals per group (inset).

One of the most intriguing features of the burials from the necropolis of Avenida 5 de Outubro was the differing orientation of their graves and in some cases the direct overlap of graves with varying orientations. Overlapping of burials was a relatively rare occurrence in medieval Islamic burials with the exception of very densely populated cities, such as Cordoba in the 10^th^ century [104]. This feature raised questions over whether there may be any meaningful difference, culturally or chronologically [91], in the way the graves were oriented. The individuals were thus assigned a classification according to their burial orientation. Three categories were thus defined: 1.) unorthodox graves that were clearly S/N oriented containing skeletons with the head to the S and feet to the N, 2.) graves that are not perfectly S/N oriented but still deviate from the canonically ‘correct’ position and contain skeletons that are oriented closer to S/N oriented than to SW/NE oriented, and 3.) canonical graves that were clearly SW/NE oriented containing skeletons with the head to the SW and feet to the NE. Fig 4 illustrates how these classifications were defined. For the ease of referring to these orientation types, these groupings are referred to in the results and discussion as: ‘Group 1’(S/N-oriented), ‘Group 2’ (Maybe S/N-oriented) and ‘Group 3’ (SW/NE-oriented). Fig 5 shows the location of the graves in the necropolis and the number of individuals in each group.

### 4.2.) Fauna

12 Faunal bones from Vila Rosa Palace, Santarém were selected for *δ*^13^C and *δ*^15^N analysis, representing both domestic and wild species (4 cows; 4 sheep/goats; 1 rabbit; 2 chickens; 1 fox). Taxa identification and anatomical diagnosis was conducted, prior to chemical analysis, at the Laboratório de Arqueologia at Universidade do Algarve [105]. Faunal material is listed in Table 2.

**Table 2:** Faunal skeletal material.

Sample	Archaeological Unit	Species	Common Name	Bone
FVRP1	Villa Rosa Palace Unit 1598	*Gallus*	Chicken	Femur
FVRP2	Villa Rosa Palace Unit 1598	*Bos Taurus*	Cow	Pelvis
FVRP3	Villa Rosa Palace Unit 1598	*Ovicaprid*	Sheep/goat	Radius
FVRP4	Villa Rosa Palace Unit 1598	*Ovicaprid*	Sheep/goat	Radius
FVRP5	Villa Rosa Palace Unit 1666	*Vulpes*	Fox	Tibia
FVRP6	Villa Rosa Palace Unit 1666	*Gallus*	Chicken	Femur
FVRP7	Villa Rosa Palace Unit 1666	*Oryctolagus*	European Rabbit	Femur
FVRP8	Villa Rosa Palace Unit 1666	*Ovicaprid*	Sheep/goat	Metatarsal
FVRP9	Villa Rosa Palace Unit 1666	*Ovicaprid*	Sheep/goat	Metatarsal
FVRP10	Villa Rosa Palace Unit 1666	*Bos Taurus*	Cow	Metatarsal
FVRP11	Villa Rosa Palace Unit 1666	*Bos Taurus*	Cow	Femur
FVRP12	Villa Rosa Palace Unit 1666	*Bos Taurus*	Cow	Femur

### 4.3.) Plants

Eight modern plant samples were collected from the various lithologic outcrops present within a 15 km radius of Santarém, in order to create a local bioavailable ^87^Sr/^86^Sr database against which the archaeological human ^87^Sr/^86^Sr data could be compared. Wild plants were selected from locations where soils were not expected to be affected by agricultural activity and/or fertilizers. Samples were collected from public lands not included within the Natura 2000 protected areas network. The location of each plant sample was recorded by GPS and is indicated in Fig 2. Plant material is listed in Table 3.

**Table 3:** Plant material.

Sample	Coordinates (WGS84)		Species	Substrate	Description
	Latitude	Longitude			
R1	39.24295	-8.67969	*Viburnum tinus*	P2	Pleiocene Calcareous
R2	39.21579	-8.68939	*Quercus*	P1	Pleiocene Sandstone and clay
R3	39.25692	-8.67792	*Ailanthus altissima*	P1	Pleiocene Sandstone and clay
R4	39.2731	-8.68799	*Quercus*	M4	Miocene calcareous, sandstone and clay
R5	39.20596	-8.70586	*Arundo donax*	alluvials	Modern
R6	39.11758	-8.71427	*Salix babylonica*	alluvials	Modern
R8	39.17685	-8.55144	*Heterotheca subaxillaris*	P3	Pleiocene Sandstone and conglomerates
R9	39.2328	-8.52477	*Quercus suber*	MP	Pleiocene clay/sandstone complex

## 5.) Methodology

### 5.1.) Collagen extraction

Approximately 500 mg of compact bone was cut with a DREMEL® rotary tool fitted with a diamond-coated disc. Pieces were then cleaned with a diamond-coated burr to remove dirt, discoloration, potential contaminants, and spongy bone. A modified Longin method [106, 107] was used to extract the bone collagen, whereby samples were each placed in 10 ml of 0.5M HCl at room temperature with overnight refrigeration, for around 14 days until demineralised. Acid was replaced after one week and samples were vortexed regularly. Once fully demineralised, acid was removed and samples were rinsed to neutrality with Milli-Q® ultrapure water. Bone samples were then placed in 10 ml of 0.125 M NaOH for 20 h at room temperature to remove organic contaminants such as humic and fulvic acids [108]. Samples rinsed to neutrality again and then placed in 10ml of 0.01 M HCl for 48 h at 70°C to facilitate gelatinisation. The solution was filtered using a 9 ml Ezee-Filter™ (60-90μm) (Elkay Laboratory Products) to remove insoluble particles. Collagen was then freeze-dried and the dry collagen was weighed to calculate collagen yield.

### 5.2.) Stable carbon and nitrogen isotope analysis

Approximately 0.7 mg of collagen was weighed into tin capsules for *δ*^13^C and *δ*^15^N analysis, conducted using an Elemental Analyser (EA Flash 2000 HT, Thermo Fisher Scientific®) coupled, via a ConFlo IV interface (Thermo Fisher Scientific®), to an isotope ratio mass spectrometer (Delta V Advantage-IRMS, Thermo Fisher Scientific®). All sample preparation and analysis of *δ*^13^C and *δ*^15^N was conducted at the HERCULES Laboratory, University of Évora. Isotope results are reported as δ values, given in parts per thousand (‰) relative to the international standards VPDB (Vienna Pee-Dee Belemnite) for carbon and AIR (Ambient Inhalable Reservoir) for nitrogen. A three point calibration [109] was used for δ^13^C composition, IAEA-CH-6 (sucrose, δ^13^C: -10.45‰), EEZ-20, (δ^13^C: -12.3‰) and IAEA-600 (caffeine, δ^13^C: -27.77‰), while for δ^15^N composition, IAEA-600 (caffeine, δ^15^N: +1‰), IAEA-N-1 (ammonium sulphate, δ^15^N: +0.43‰) and IAEA-N-2 (ammonium sulphate, δ^15^N: +20.3‰) were used for calibration. L-Alanine (δ^13^C: -18.39‰; δ^15^N: +0.91‰) was used as an in-house check standard interspersed during each run. Measurement uncertainty was calculated according to the method presented by Szpak *et al*. (2017) and using the Microsoft Excel spreadsheet templates provided in that paper [109] (See S1 Table). Standard uncertainty (*u*_*c*_) is estimated as a pooled standard deviation of precision (u(R_w_) ±1σ) while accuracy (*u(bias*) ±1σ) is calculated using the variability in calibration standards and check standards. Precision (u(R_w_)) was determined to be ± 0.12‰ for δ^13^C and ±0.09‰ for δ^15^N. Accuracy (*u(bias*)) was determined to be ± 0.17‰ for δ^13^C and ± 0.23‰ for δ^15^N and the total uncertainty over 7 analytical sessions was estimated to be ± 0.21‰ for δ^13^C and ± 0.25‰ for δ^15^N.

### 5.3.) Stable sulphur isotope analysis

Sulphur isotope analysis was conducted at SIIAF, University of Lisbon, using an IsoPrime mass spectrometer. Approximately 6mg of collagen was placed into capsules with additional 1mg V_2_O_5_ and combusted with a pulse of oxygen. δ^34^S was calibrated using the inorganic international standards NBS 127 (+20.3‰), IAEA S1 (-0.3‰), and B2155 Protein (+6.32‰) as an internal standard. Precision (u(R_w_)) was determined to be ± 0.1‰, while accuracy (*u(bias*)) was ± 0.96‰, providing a total uncertainty of ± 0.97‰ for δ^34^S over 2 analytical sessions (see S1 Table). Stable sulphur isotope results are reported as δ values, given in parts per thousand (‰) relative to VCDT (Vienna- Canyon Diablo Troilite).

### 5.4.) Stable carbon and oxygen isotope analysis

Prior to sampling, the preservation of bone apatite was assessed using ATR-FTIR, to ensure suitability for δ^13^C and *δ*^18^O analysis (See S2 Text for methodology). Subsequently, the outer surface of the tooth enamel and bone samples were cleaned with a DREMEL® rotary tool fitted with a diamond coated burr to remove soil particles and contaminants. Approximately 15 mg of bulk tooth crown enamel/bone powder was collected and ground with an agate mortar and pestle to obtain a homogenous sample material. The powder was bleached in 1 ml NaOCl (2–3 vol%) for 24h at room temperature to remove organic matter and contaminants. Samples were then rinsed to neutrality with Milli-Q® ultrapure water and placed in 1 ml of 1M acetic acid buffered with Li-acetate solution for 12 h (for tooth enamel) or 24 h (for bone powder) to remove any adsorbed or diagenetic carbonates [110–112]. Samples were rinsed again to neutrality and dried overnight at 70°C. Subsequently, the stable oxygen and *δ*^13^C data were determined for the carbonate fraction of the samples. For that purpose, approx. 800–850 μg was dissolved in He-flushed borosilicate exetainers at 72°C using a water-free phosphoric acid. The released CO_2_ gas was then measured in a Continuous Flow- Isotope Ratio Mass Spectrometer (Thermo Fisher Scientific® 253) coupled to a GasBench II at the Institute of Geosciences, University of Mainz. *δ*^13^C and *δ*^18^O were calibrated with a 1-point calibration using the Carrara Marble (δ^13^C: +2.01‰; δ^18^O: -1.91‰) because the isotope data of the samples was very close to that of the Carrara Marble. Results are given in parts per thousand (‰) relative to VPDB. The average precision error (1σ) was found to better than 0.04‰ for δ^13^C and 0.04‰ for δ^18^O and the accuracy was within 0.01‰ for both isotope systems.

The *δ*^18^O values (relative to VPDB) were first converted to the VSMOW-scale, using the equation (see Eq 1) established by Coplen *et al*. [113]:

$$\delta^{18}O_{VSMOW}=1.03091\;x\;\delta^{18}O_{VPDB}+30.91$$ (1)

The conversion of carbonate to phosphate oxygen isotope values (*δ*^18^O_c_ to *δ*^18^O_p_) was done using the equation (see Eq 2) proposed by Chenery *et al*. [44] while the estimated drinking water oxygen isotope values (*δ*^18^O_DW_) were calculated using the equation (see Eq 3) established by Chenery *et al*. [44] which in turn was based on the drinking water equation of Daux *et al*. [43].


$$\delta^{18}O_{p}=1.0322\;x\;\delta^{18}O_{c}-9.6849$$ (2)

$$\delta^{18}O_{DW}=1.590\;x\;\delta^{18}O_{c}-48.634$$ (3)

### 5.5.) Radiogenic strontium isotope analysis

For human tooth samples, the enamel surface was cleaned with a DREMEL® rotary tool fitted with a diamond coated burr and around 20 mg of bulk enamel powder was collected from the tooth crown, with care taken to avoid any sand or dirt particles. Enamel was ground with an agate mortar and pestle, then placed into 1.2 ml acetic acid buffered with Li-acetate solution for 12h. Sampled were then rinsed to a neutral pH with Milli-Q® ultrapure water and dried overnight at 70°C. Plant samples were rinsed with Milli-Q® ultrapure water to remove any exogenous dust or contamination, dried at 50°C for 48h, placed into quartz crucibles and ashed for 12h at 550°C in a muffle furnace. Approximately 20 mg of plant ash was collected and placed in microtubes. Sample digestion and strontium extraction for tooth enamel powder and plant ash samples was conducted at the Isotope Geology Laboratory of the University of Aveiro. All samples were digested using HNO_3_ acid, and in plant samples an additional step of digesting with H_2_O_2_ was used. After evaporation to dryness the samples were dissolved in HCL 6.2M and dried again. All reagents used were sub-boiling distilled, and the water produced by a Milli-Q Element (Millipore) apparatus. Columns filled with Sr-Resin (Sr-B10-S, 50-100mesh, TrisKem International) were used for purified strontium from the remaining elements. Then the extracted Sr was loaded onto Ta filaments with H_3_PO_4_ and the isotopic composition measured with a VG Sector 54 TIMS in dynamic mode with ^88^Sr peak measurements at 1–2 V. Typical runs consisted of acquisition of 60 isotopic ratios and the analytical data were corrected for mass fractionation using the exponential law relative to ^88^Sr/^86^Sr = 0.1194. The standard reference material NIST SRM 1400 (bone ash) was concomitantly measured and provided a value of ^87^Sr/^86^Sr = 0.713160 ± 0.000021, within the GEOREM [114] database range of 0.7131–0.7134. In addition, the mass spectrometry standard SRM 987 gave, in the two periods of experimental work, mean values of ^87^Sr/^86^Sr = 0.710240 ± 31 and 0.710266 ± 11, (conf. limit 95%, N = 13 and 12, respectively).

### 5.6.) Ancient DNA analysis

Ancient DNA (aDNA) molecules were extracted from the petrous bone using a standard method suited for degraded fragments [115] and partially UDG-treated double-stranded DNA libraries [116] were generated. DNA extraction and sequencing library preparation steps were performed at the Australian Centre for Ancient DNA (ACAD)’s ultra-clean laboratory facilities, while post-amplification experiments were completed in standard molecular biology laboratories at the University of Adelaide following rigorous laboratorial procedures to minimise contamination and ensure high standards of quality for the genetic data. Shotgun sequencing was performed on an Illumina NovaSeq instrument at the Kinghorn Centre for Clinical Genomics (Sydney, NSW, Australia). A detailed methodology is provided in S1 Text.

Genetic sex determination was assigned using X- and Y-ratios, Rx ratios [117] and X chromosome read dosage (Mx) [118] (see S1 Text). A relatively low number of authentic ancient DNA fragments was initially obtained and only 3 libraries passed quality thresholds to undergo re-amplification and enrichment for DNA sequences overlapping 46k informative SNPs located on the Y chromosome and the whole mitochondrial genome using the myBaits Expert Human Affinities Prime Plus Kit by Daicel Arbor Biosciences (Ann Harbor, MI, USA). The enriched libraries were sent to the Kinghorn Centre for Clinical Genomics (Sydney, NSW, Australia) for sequencing on an Illumination NovaSeq instrument. Mitochondrial haplogroups were called using HAPLOFIND [119]. To obtain Y-chromosome haplotypes for the male individual, a VCF file selecting only the Y chromosome sites was input into yhaplo v1.1.2. [120].

### 5.7.) Reconstruction of solar position

In order to test some theories regarding the position of the sun in different seasons and how this may affect the ability of the gravediggers to estimate of the direction of Mecca, the online model SunCalc [121] was employed. Various times of the year including 1 January (for midwinter) and 20 June (for midsummer) were entered into the model, and the position of the sun at both sunrise and sunset from the location of Santarém was observed.

### 5.8.) Statistical analysis and data management

The statistical comparison of isotope results, to assess groups within the population, was conducted using the online statistics calculator “Statistics Kingdom, 2017” [122]. Non-parametric Kruskal-Wallis tests (when there were three groups i.e. Grave Orientation) and Mann-Whitney *u* tests (when there were two groups i.e. Sex) were used for non-normally distributed data to assess the mean ranks between the groups, whereby the shape of distribution within each group is assumed to be equal. In the cases where a significant difference between groups was found, a Post-Hoc Dunn’s test with a Bonferroni correction was used to identify which pairs were significantly different. A Levene’s test was used to assess if there was a statistically significant difference of variability between the groups. These results are provided in S3 Text. All isotope data from this study has been made available on the IsoArcH database [123, 124], DOI 10.48530/isoarch.2023.007 and is accessible via the URL https://isoarch.eu/datasets/2023-007/. This dataset is available under the Creative Commons BY-NC-SA 4.0 license.

## 6.) Results

### 6.1.) Bone collagen and apatite preservation

The bone collagen samples presented here (Table 4) had C:N ratios within the acceptable range [125, 126] for good preservation (humans: 3.1 ± 0.1; fauna: 3.1 ± 0.0) and overall had good collagen yields (humans: 10 ± 3%; fauna: 9 ± 4%), with the exception of HS-1979, HS-918, HS-923, HS-958, HS-1027 and HS-1061 which yielded no collagen. The carbon (humans: 39.2 ± 9.4%; fauna: 39.8 ± 2.7%) and nitrogen (humans: 14.8 ± 3.5%; fauna: 14.9 ± 1.0%) amounts indicated good collagen preservation [127], with the exception of one faunal sample (FVRP6- chicken) and the humans HS-819, 955-H, HS-2079, HS-2215, HS-2275, and HS-2316 who were excluded from further analysis and interpretation. The samples HS-2003 and FVRP12 were excluded from comparison due to instrument error in their analysis. This left a total number of 45 human samples and 10 faunal samples with comparable bone collagen results. Regarding the sulphur, modern bones typically have %S values that range from 0.15 to 0.35% as well as C:S and N:S ratios that range from 300 to 900 and 100 to 300 respectively [128, 129]. With the exception of the previously mentioned human samples and HS-2170, and the faunal samples FVRP6 and FVRP12, which were excluded from further comparison, the bone collagen sample values fell within these ranges and were considered to be reliable. Additionally, the preservation of bone apatite for a selection of individuals was assessed using ATR-FTIR. The methodology and results are provided in S2 Text. These results indicated that the bones were not significantly altered by diagenetic processes and thus any *δ*^13^C_ap_ values obtained from them are likely to reflect in vivo carbon in the bone apatite.

**Table 4:** Results-Bones.

Sample	Grave Orientation Group	Collagen Yield (wt %)	%C	%N	C/N	δ^13^C_col_ (‰) VPDB	δ^15^N (‰) AIR	δ^13^C_ap_ (‰) VPDB	%S	C/S	N/S	δ^34^S (‰) VCDT
**HS-557**	1	11.7	40.9	15.6	3.1	-19.1	10.3	-12.2				
**HS-561**	1	9	42.6	16.2	3.1	-19.3	9.1	-12.9	0.30	378.6	123.2	12.2
**HS-931**	1	6.6	41.9	15.0	3.3	-19.9	10.6	-12.0				
**955-H**	1	8.6	*23*.*8*	*8*.*8*	3.1	*-18*.*6*	*11*.*1*		0.21	301.9	*96*.*2*	*11*.*0*
**HS-1156**	1	14.6	40.2	15.2	3.1	-18.8	10.5		0.28	382.6	124.0	9.3
**1442-F**	1	12.3	41.8	15.8	3.1	-19.2	10.6		0.25	446.0	144.3	10.1
**1647-F**	1	10.9	43.5	16.5	3.1	-19.0	10.6	-12.3	0.26	446.1	145.4	8.8
**HS-1865**	1	5.6	42.5	16.2	3.1	-19.1	9.7	-11.7	0.32	354.1	116.0	12.0
**HS-1985**	1	8.6	42.6	16.3	3.0	-19.3	9.8	-12.6	0.31	366.6	120.4	10.7
**HS-2003**	1	11.6	*84*.*3*	*31*.*1*	3.2	*-18*.*2*	*10*.*3*	-11.8	0.30	*749*.*7*	*236*.*7*	*8*.*8*
**HS-2242**	1	13.1	42.6	16.0	3.1	-19.1	8.4	-12.4	0.30	378.5	122.2	12.1
**HS-2271**	1	4.6	40.9	14.9	3.2	-18.7	11.8	-10.4	0.25	436.7	136.2	10.5
**HS-2273**	1	9.8	28.9	10.7	3.1	-19.1	10.9	-11.8	0.18	428.2	136.1	13.9
**HS-2316**	1	8.5	27.9	*10*.*3*	3.2	*-18*.*9*	*9*.*8*	-12.9	0.22	338.3	106.9	*12*.*8*
**2334-F**	1	10	42.8	16.2	3.1	-19.2	11.1		0.30	380.7	123.5	13.6
**HS-612**	2	9.9	43.6	16.6	3.1	-18.8	10.1	-12.4	0.29	400.9	131.0	11.2
**639-F**	2	5.6	41.5	15.8	3.1	-18.8	10.1	-12.0	0.28	395.4	128.9	10.2
**HS-761**	2	9.9	39.7	15.3	3.0	-19.0	11.0	-11.0	0.28	377.8	124.7	9.9
**HS-921**	2	13.6	42.2	16.2	3.0	-17.0	9.1	-10.7	0.20	562.6	185.2	11.0
**1092-F**	2	9.7	43.4	16.4	3.1	-18.9	11.5	-12.1	0.25	462.7	150.0	10.3
**HS-1251**	2	5.3	40.5	15.2	3.1	-19.2	10.5	-12.5	0.28	385.5	124.5	10.0
**HS-1491**	2	9.4	43.0	15.8	3.2	-18.8	11.3	-12.1	0.31	370.1	116.7	7.4
**HS-1584**	2		42.2	16.1	3.1	-18.6	9.4	-11.2				
**HS-1604**	2	13.5	38.6	14.5	3.1	-18.3	10.3		0.22	467.4	150.4	11.0
**2075-F**	2	10.3	42.4	16.1	3.1	-19.3	11.9	-12.8	0.27	418.8	136.0	9.9
**HS-2128**	2	9.2	42.8	16.3	3.1	-18.6	9.1	-12.7	0.31	368.2	120.5	10.4
**HS-2275**	2	12.4	25.5	*9*.*4*	3.2	*-19*.*2*	*11*.*1*	-10.7				
**HS-559**	3	7.4	41.5	15.4	3.1	-19.5	9.7	-11.8	0.29	381.4	121.5	16.0
**HS-811**	3	9.9	41.4	15.4	3.1	-19.4	12.4	-11.1	0.24	459.6	146.3	8.9
**HS-819**	3	11.2	*16*.*1*	*5*.*9*	3.2	*-18*.*8*	*10*.*3*	-11.5	0.18	*237*.*9*	*74*.*8*	*14*.*0*
**HS-867**	3	13.9	28.5	10.4	3.2	-19.0	11.8	-12.3	0.19	400.4	125.7	10.8
**HS-956**	3	11.1	43.1	16.4	3.1	-19.2	9.8	-12.7	0.29	396.6	129.3	9.8
**HS-1103**	3	2.3	40.1	15.0	3.1	-19.1	12.1	-11.0	0.27	396.4	126.9	7.3
**HS-1135**	3	8.9	41.6	15.6	3.1	-18.2	9.5	-11.7	0.25	443.6	142.2	12
**HS-1325**	3	11	43.6	16.9	3.0	-19.1	9.7	-12.4	0.34	342.0	113.3	9.7
**HS-1480**	3	3.4	42.0	16.0	3.1	-19.1	9.3	-11.1	0.31	361.6	118.1	12.5
**HS-1619**	3	11.7	39.1	14.9	3.1	-19.0	10.1	-12.3	0.31	336.1	110.2	11.3
**HS-1622**	3	7.8	29.0	10.8	3.1	-19.5	9.0	-11.7				
**1782-F**	3	14.2	42.0	15.9	3.1	-18.7	9.8	-12.4	0.26	431.1	139.7	13.2
**HS-1794**	3		42.1	16.0	3.1	-19.3	11.8	-11.6				
**1976-F**	3	7.5	41.9	15.9	3.1	-19.1	10.4	-12.1	0.25	447.2	145.5	15.0
**HS-2079**	3	12.4	*23*.*1*	*8*.*6*	3.1	*-19*.*4*	*9*.*5*	-12.9	0.16	385.5	122.6	*12*.*0*
**HS-2082**	3	11.5	42.3	15.8	3.1	-18.5	11.3	-12.0	0.24	470.0	150.4	10.5
**HS-2170**	3	4	40.2	15.3	3.1	-18.9	11.2	-11.6	*0*.*37*	*289*.*4*	*94*.*6*	*9*.*3*
**HS-2181**	3	13.6	41.8	15.6	3.1	-18.9	11.5	-13.4	0.29	384.6	122.8	7.8
**HS-2189**	3	11.9	42.0	16.0	3.1	-19.3	10.0	-12.5	0.31	361.3	117.7	9.7
**HS-2215**	3	12.2	*22*.*4*	*8*.*4*	3.1	*-19*.*1*	*9*.*3*	-11.7	*0*.*14*	426.1	137.6	*10*.*9*
**HS-2320**	3	10.5	36.4	14.2	3.0	-18.2	9.7	-12.3	0.28	346.3	115.8	10.9
**HS-2356**	3	15.3	29.1	11.5	3.0	-18.6	10.7		0.17	456.9	154.0	10.6
**HS-2358**	3	7.3	42.8	16.2	3.1	-19.3	10.2	-9.9	0.32	356.4	115.7	9.3
**HS-2361**	3	10.8	43.3	16.7	3.0	-19.0	9.0	-11.2	0.30	385.0	127.1	11.6
**HS-2423**	3	13.2	31.0	12.3	2.9	-19.0	9.2		0.22	375.7	127.5	4.9
**Human Mean**		**9.9 ± 3.1**	**39.1 ± 9.4**	**14.8 ± 3.5**	**3.1 ± 0.1**	**-19.0 ± 0.5**	**10.4 ± 1.0**	**-11.9 ± 0.7**	**0.3 ± 0.0**	**400.7 ± 49.3**	**130.1 ± 15.8**	**10.6 ± 2.1**
FVRP1		5.6	41.1	15.3	3.1	-16.9	9.1		0.29	378.4	120.6	16.3
FVRP2		13.3	41.6	15.7	3.1	-20.7	6.7		0.23	481.9	156.1	13.3
FVRP3		13.2	36.8	13.9	3.1	-19.6	5.5		0.21	466.8	151.5	9.6
FVRP4		12.8	33.5	12.7	3.1	-20.9	8.0		0.21	425.4	138.1	12.5
FVRP5		6.6	41.1	15.5	3.1	-18.1	8.9		0.28	391.3	126.2	10.8
FVRP6		*3*.*67*	*0*.*9*	*0*.*4*	*2*.*7*				0.33	*7*.*0*	*2*.*6*	10.1
FVRP7		2.4	38.8	14.6	3.1	-20.2	5.6		0.3	345.3	110.9	16.2
FVRP8		12.9	41.3	15.6	3.1	-20.5	4.6		0.29	379.3	122.6	12.8
FVRP9		5.7	40.8	15.4	3.1	-20.6	5.7		0.26	418.9	135.3	9
FVRP10		12.6	42.2	15.3	3.2	-20.4	6.2		0.26	433.1	134.6	13.3
FVRP11		6.2	40.9	15.3	3.1	-19.7	6.8		0.26	419.7	134.7	12.8
FVRP12		8.0	*93*.*7*	*35*.*2*	3.1	-18.6	7.8					

### 6.2.) Stable carbon (bone collagen and apatite) and nitrogen isotope results

**Fig 6:**
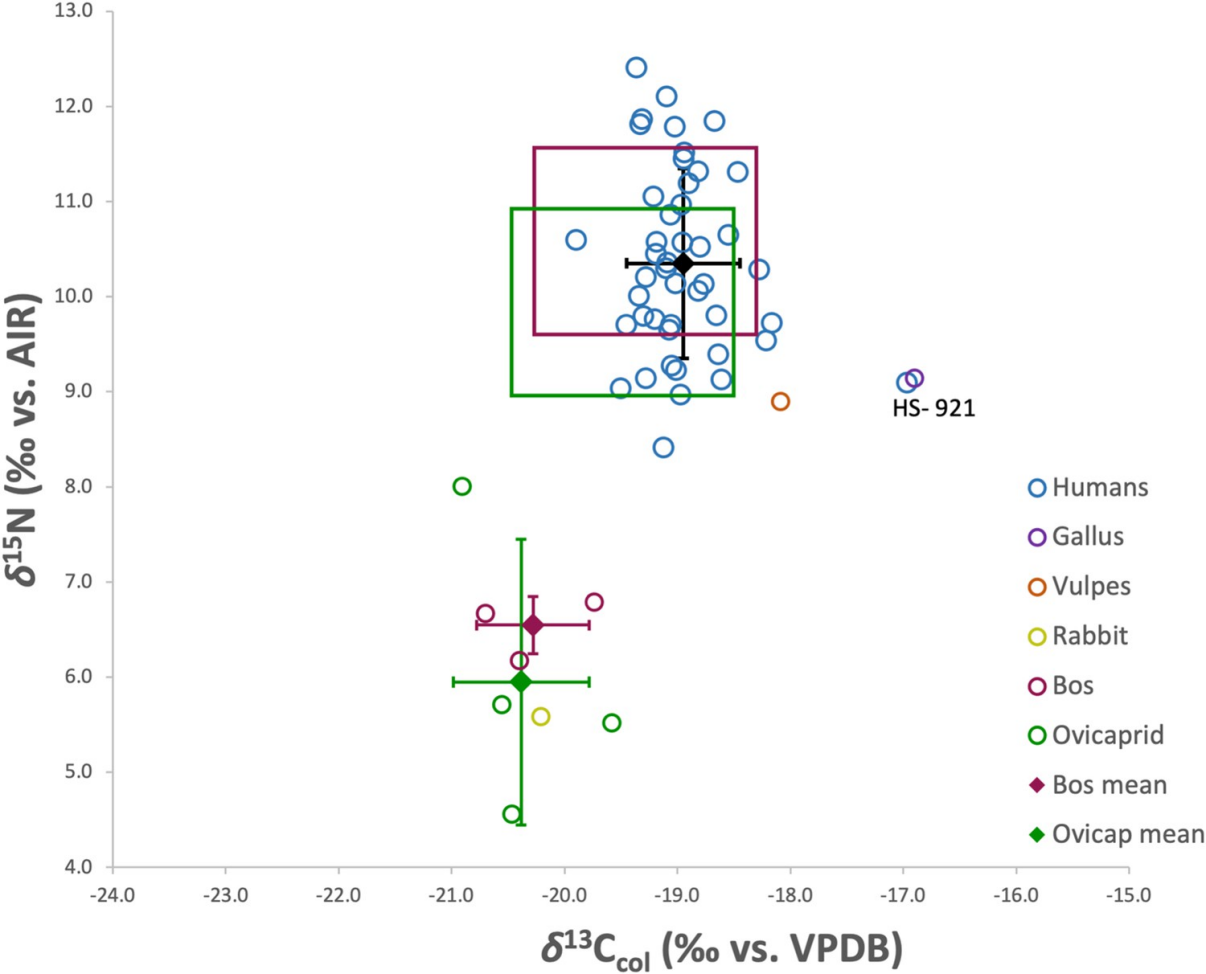
Human and fauna *δ*^15^N and *δ*^13^C_col_. Squares represent expected range of +1 trophic level (+0–2‰ in *δ*^13^C_col_; +3–5‰ in *δ*^15^N) increase over mean *Bos* and Ovicaprid values, respectively.

The analysis of *δ*^13^C and *δ*^15^N in the human bone collagen (n = 45) resulted in a mean *δ*^13^C_col_ value of -19.0 ± 0.5‰ (ranging from -19.9‰ to -17.0‰) and a mean *δ*^15^N value of 10.4 ± 1.0‰ (ranging from 8.4‰ to 12.4‰). Regarding the faunal bone collagen isotope values, *δ*^13^C_col_ ranged from -20.9‰ to -16.9‰ and *δ*^15^N values ranged from 4.6‰ to 9.1‰ (Table 4). The animals have *δ*^13^C_col_ values that are consistent with a C_3_ plant-based diet, with the exception of one chicken (FVRP1) which may have been partially fed on C_4_ grain (i.e., millet), and the fox (FVRP5) which may have consumed food from various wild sources as well as human food scraps. As Fig 6 illustrates, the human *δ*^13^C_col_ values appear to be quite homogenous, with only one clear outlier (HS-921), and overall this population falls within the expected trophic level increase of 0–2‰ in *δ*^13^C_col_ [130] over the domestic terrestrial herbivores (*Bos* and ovicaprids). Concerning the *δ*^15^N values of the fauna, the highest belong to the fox (FVRP5) and chicken (FVRP1), both omnivorous species that are trophically more similar to humans. The *Bos* (n = 3) *δ*^15^N values are quite homogenous, while the ovicaprids (n = 4) are more diverse, possibly reflective of the more varied grazing patterns of sheep and goats compared to cows. Applying an estimated trophic offset of 3–5‰ [130] to the mean *δ*^15^N values of the domestic herbivores, it appears that the main protein components in the diet of this population likely came from local, C_3_ plant-fed, domestic herbivores.

**Fig 7:**
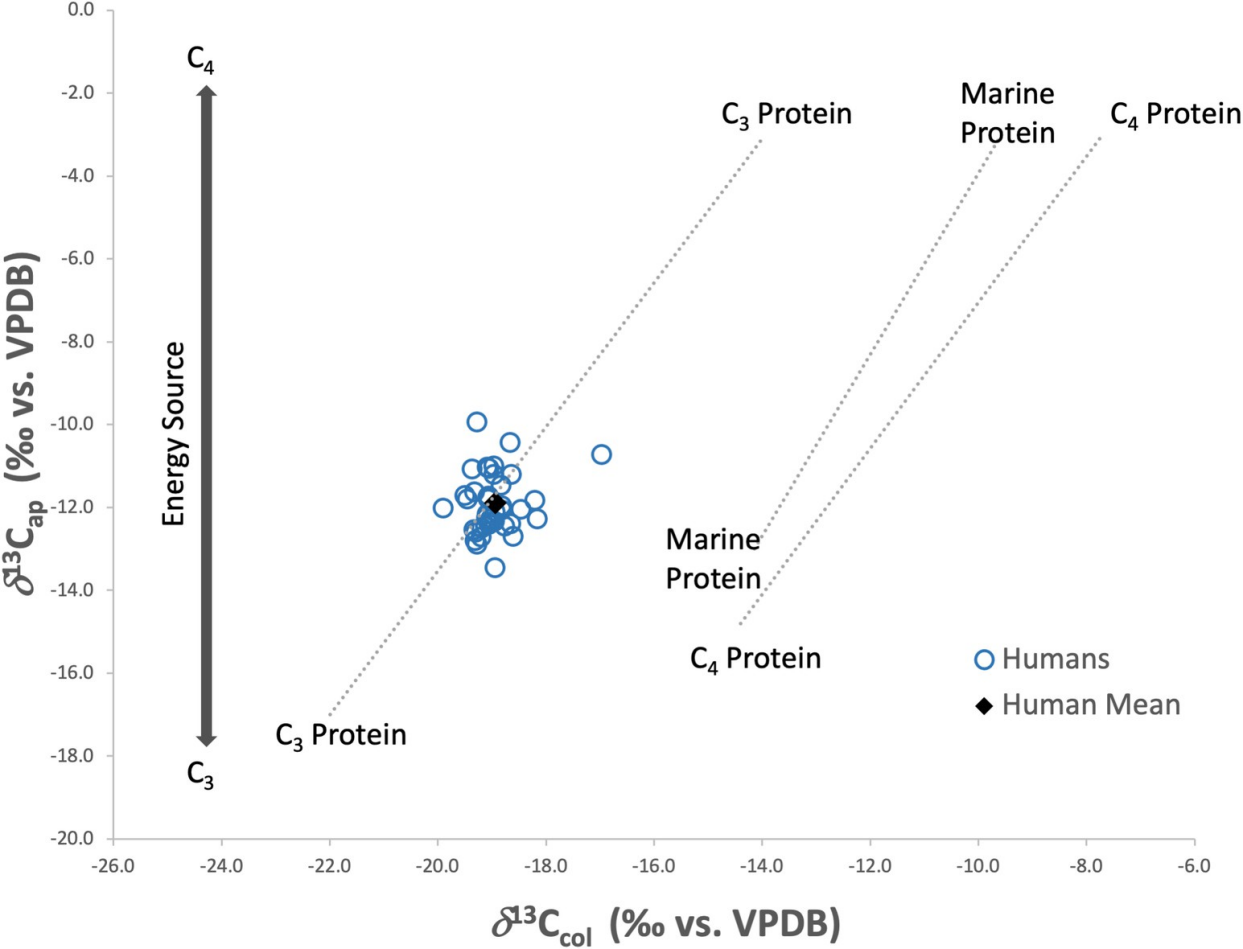
Human *δ*^13^C_col_ and *δ*^13^C_ap_ plotted against regression lines [131] indicating main protein and energy sources in diet.

The stable carbon isotopes measured in the human bone apatite (*δ*^13^C_ap_) yielded a mean value of -11.9 ± 0.7‰, ranging from -13.4‰ to -9.9‰. Comparing *δ*^13^C_col_ and *δ*^13^C_ap_ values for the bones (Fig 7), the overall homogeneity of diet for this population is still apparent. When plotted against a model developed by [131] to evaluate both protein and energy (whole diet) inputs, the *δ*^13^C_col_ values are consistent with C_3_ based protein inputs for the population (except HS-921 who appeared to have a higher C_4_ input) while the *δ*^13^C_ap_ values indicate that there was a dominance of C_3_ with some mixing of C_4_ based energy sources in the diets of these individuals.

### 6.3.) Stable sulphur isotope results

**Fig 8:**
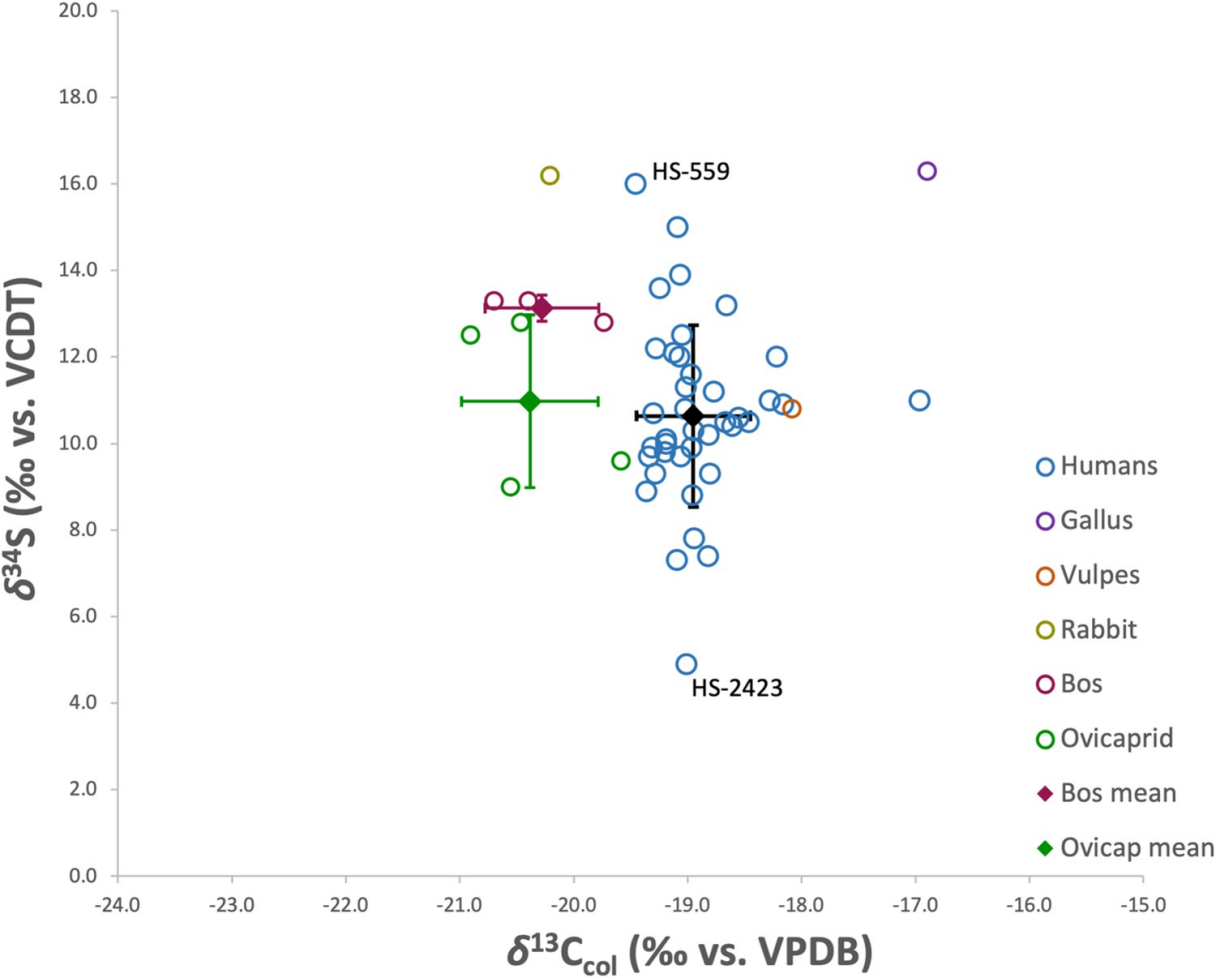
Human and fauna *δ*^34^S and *δ*^13^C.

The analysis of *δ*^34^S in the bone collagen of the Santarém humans (n = 46) yielded an average *δ*^34^S value of 10.6 ± 2.1‰, ranging from 4.9‰ to 16‰. The faunal bone collagen yielded an average *δ*^34^S value of 12.7 ± 2.4‰, ranging from 9‰ to 16.3‰. The humans have similar *δ*^34^S values to the fauna with the exception of four individuals with lower *δ*^34^S values (HS-2423; HS-1103; HS-1491 and HS-2181) (see Fig 8).

### 6.4.) Tooth enamel stable carbon and oxygen isotope results

In addition to bone collagen and apatite, stable carbon isotopes were also analysed in the tooth enamel (*δ*^13^C_en_) of 31 teeth, from 22 individuals (9 individuals were selected for multi-tooth analysis) in order to assess whole diet inputs during childhood. These values are all given in Table 5. These results yielded a mean *δ*^13^C_en_ value of -11.9 ± 1.0‰, ranging from -14.0‰ to -9.7‰. Of the individuals for which multiple teeth were analysed, the differences in *δ*^13^C_en_ values ranged from 0.2‰ to 2.1‰, but no consistent pattern could be identified of either increasing or decreasing values *δ*^13^C_en_ amongst individuals that might indicate changes in diet during childhood. Stable oxygen isotopes were analysed to assess the mobility of the individuals. A mean *δ*^18^O_DW_ value of -5.7 ± 1.0‰ was calculated from the tooth enamel of these individuals, ranging from -8.6‰ to -4.4‰. Concerning the individuals for which two teeth were sampled, the M1 was more enriched than the M3 in only two cases (2334-F and HS-1584), with a difference of +0.1‰ and 0.3‰, respectively. For the other individual in which both an M1 and M3 were sampled, the M1 had more depleted *δ*^18^O_DW_ values (HS-2079: M1 = -5.8‰, M3 = -5.0‰). If all *δ*^18^O_c_ values for M1s were corrected by -1‰, a conservative estimate using the breastfeeding enrichment range of 0.5–1.2‰ given in [132, 133] and the *δ*^18^O_DW_ values recalculated, they would be as follows: 955-H = -9.0‰; HS-1584 = -7.3; HS-1092 = -6.4‰; HS-639 = -6.1‰; HS-2079 = -7.4‰; HS-559 = -7.5‰; 2334-F = -6.7‰ and HS-1976 = -6.9‰.

**Table 5:** Results-tooth enamel.

Sample	Tooth	Grave Orientation Group	δ^13^C_en_ (‰) VPDB	δ^18^O_c_ (‰) VPDB	δ^18^O_c_ (‰) VSMOW	δ^18^O_p_ (‰) VSMOW	δ^18^O_DW_ (‰) VSMOW	^87^Sr/^86^Sr
HS-931	PM2	1	-12.1	-3.7	27.1	18.6	-5.6	
	M3		-11.1	-3.2	27.6	18.8	-4.8	0.709685
955-H	M1	1	-12.1	-4.8	26.0	17.1	-7.4	0.710225
HS-1027	M3	1	-12.4	-4.5	26.3	17.4	-6.9	0.711843
HS-1061	M2	1	-12.3	-3.2	27.6	18.8	-4.8	0.708903
HS-1156	PM2	1	-9.7	-3.8	27.0	18.2	-5.7	
	M2		-9.9	-3.4	27.4	18.6	-5.0	0.711775
1442-F	M2	1	-12.3	-5.4	25.3	16.4	-8.4	
	M3		-10.4	-5.5	25.2	16.3	-8.6	0.710115
1647-F	M3	1	-12.2	-3.2	27.6	18.8	-4.8	0.709157
HS-2003	M2	1	-12.3	-3.6	27.2	18.4	-5.4	0.711109
HS-2242	M2	1	-12.1	-4.2	26.5	17.7	-6.4	0.709039
2334-F	M1	1	-14.0	-3.4	27.4	18.6	-5.1	0.710035
	M3		-11.9	-3.5	27.3	18.5	-5.2	
HS-612	M2	2	-12.1	-3.7	27.1	18.3	-5.6	0.711708
639-F	M1	2	-12.3	-3.0	27.8	19.0	-4.4	0.709096
1092-F	M1	2	-12.0	-3.2	27.6	18.8	-4.8	0.712659
HS-1251	M3	2	-12.1	-3.7	27.1	18.3	-5.5	0.709308
HS-1584	M1	2	-11.3	-3.8	27.0	18.2	-5.6	0.708678
	M3		-10.6	-3.9	26.9	18.0	-5.9	
2075-F	M3	2	-10.6	-3.8	27.0	18.2	-5.7	0.711152
HS-2128	PM1	2	-10.1	-4.0	26.8	18.0	-6.0	
	M3		-11.4	-4.7	26.1	17.2	-7.1	0.711188
HS-559	M1	3	-13.2	-3.9	26.9	18.1	-5.8	0.708103
	M2		-12.6	-3.0	27.8	19.0	-4.4	
HS-956	M2	3	-12.5	-3.4	27.4	18.6	-5.1	0.708523
1976-F	M1	3	-13.3	-3.5	27.3	18.5	-5.3	0.710758
	M2		-12.7	-3.1	27.7	19.0	-4.5	
HS-2079	M1	3	-12.9	-3.8	27.0	18.1	-5.8	0.709231
	M3		-12.7	-3.3	27.5	18.7	-5.0	
HS-2320	M3	3	-12.5	-3.6	27.2	18.4	-5.4	0.709863
**Mean**			**-11.9 ± 1.0**	**-3.8 ± 0.6**	**27.0 ± 0.7**	**18.2 ± 0.7**	**-5.7 ± 1.0**	**0.710098 ± 0.001259**

### 6.5.) Tooth enamel radiogenic strontium isotope results

**Fig 9:**
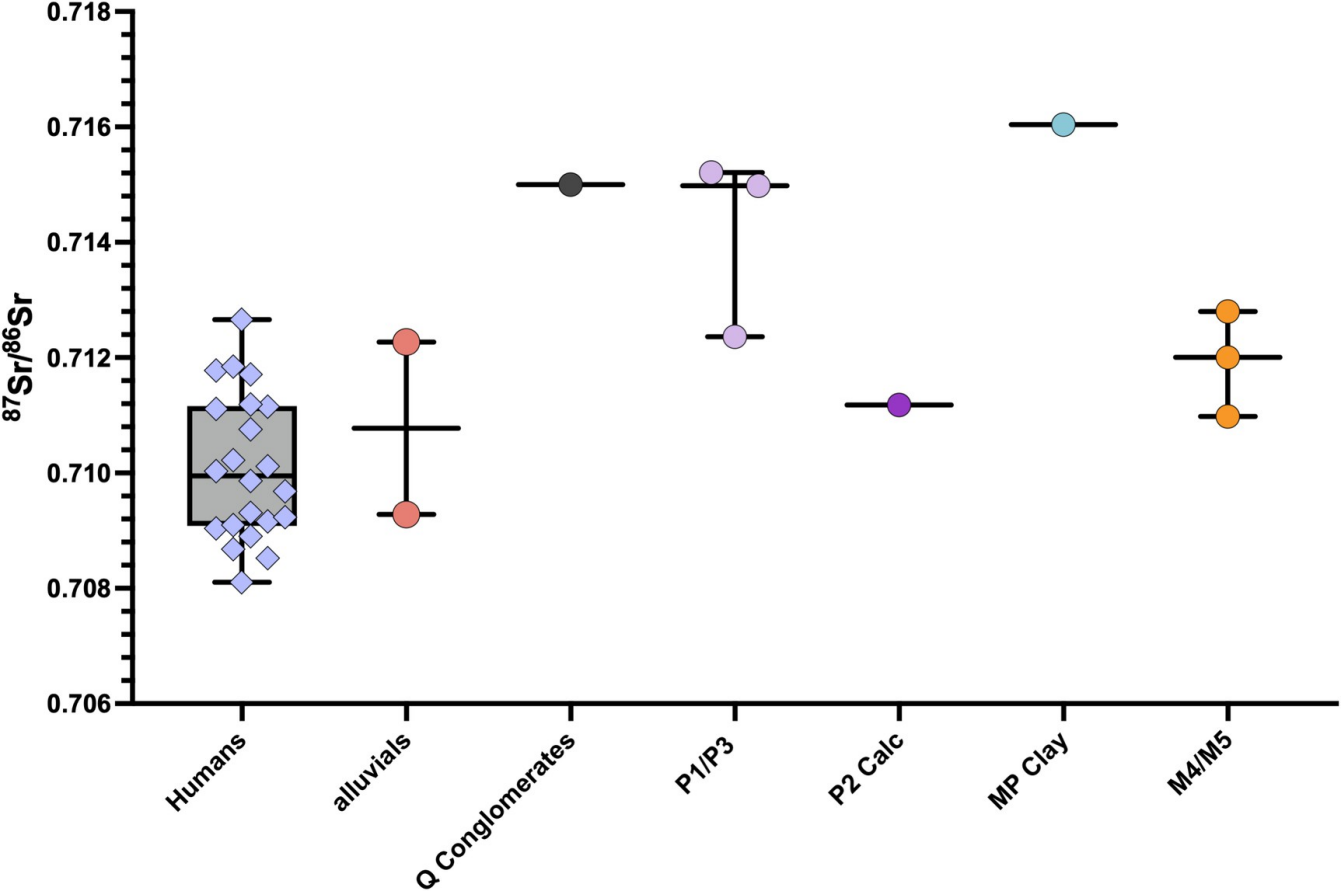
Human ^87^Sr^/86^Sr results compared to bioavailable ^87^Sr^/86^Sr for Santarém (plant ash samples from this study and [61].

The ^87^Sr/^86^Sr ratios of the plants collected from various lithologies around Santarém are provided in Table 6. The estimated local bioavailable ^87^Sr/^86^Sr range, based on these plant samples as well as the plant samples (PT16-104; PT16-109; PT16-110) collected close to Santarém in a recent study [61] is considered as 0.709–0.716 for the purpose of this study. The ^87^Sr/^86^Sr ratios obtained from the human tooth enamel, given in Table 5 gave a mean value of 0.710098 ±0.001259, ranging from 0.708103 to 0.712659. When the plant sample ^87^Sr/^86^Sr ratios are grouped according to the geological substrate from which they were sampled (see Figs 2, 9 and Table 3), the human range shows consistency with food sourced on the alluvial plains, the Pleiocene plateau (P2) upon which Santarém is located and the Miocene limestone outcrops to the west of the Tagus river. The plants sampled from the sandstone, clay and conglomerate outcrops (Q/P3/MP) to the east of the river, however, yielded ^87^Sr/^86^Sr ratios higher than any of the humans, therefore they are unlikely to have consumed high proportions of food grown in this region. A few individuals also yielded ^87^Sr/^86^Sr ratios lower than 0.709 which is below the measured regional range.

**Table 6:** Results-Plant ash.

Sample	Coordinates		^87^Sr/^86^Sr	Substrate	Description	Reference
R1	39.24295	-8.67969	0.711843	P2	Pleiocene Calcareous	This Study
R2	39.21579	-8.68939	0.715212	P1	Pleiocene Sandstone and clay	This Study
R3	39.25692	-8.67792	0.712365	P1	Pleiocene Sandstone and clay	This Study
R4	39.2731	-8.68799	0.712004	M4	Miocene calcareous, sandstone and clay	This Study
R5	39.20596	-8.70586	0.71227	alluvials	Modern	This Study
R6	39.11758	-8.71427	0.709279	alluvials	Modern	This Study
R8	39.17685	-8.55144	0.714982	P3	Pleiocene Sandstone and conglomerates	This Study
R9	39.2328	-8.52477	0.716042	MP	Pleiocene clay/sandstone complex	This Study
**Mean**			**0.71247 ± 0.00416 (2σ SD)**			
PT16-104	39.1324	-8.6086	0.71500	Q2	Pleistocene conglomerates	(61)
PT16-109	39.2922	-8.8092	0.71280	M5	Miocene calcareous, sandstone and clay	(61)
PT16-110	39.2821	-8.8014	0.71098	M5/M4	Miocene calcareous, sandstone and clay	(61)

### 6.6.) Ancient DNA results

Ancient DNA analysis was performed on a subset of individuals to investigate aspects of their biological ancestry, in particular whether uniparental markers could inform on likely ancestral origins outside Iberia. These results are provided in Table 7.

**Table 7:** Ancient DNA results.

Sample Information	Individual	HS-1584	2334-F	1092-F	1647-F	HS-931	955-H
	ACAD ID	24169	24170	24171	24172	24173	24174
	Library ID	LP123_9	LP114_10	LP114_11	LP114_12	LP114_13	LP114_14
**Shotgun sequencing statistics**	Sequenced reads filtered by read length and sequencing quality	846,361	13,035,351	14,476,581	12,677,013	14,642,455	15,201,514
	Mapped reads	12.236	1,262,013	45,692	29,615	467,916	25,088
	Endogenous DNA (%) Post-Mapping	1.45	9.68	0.32	0.23	3.2	0.17
	Reads filtered by mapping quality	8,456	905,865	17,911	17,231	342,605	15,040
	Deduplicated reads	8,359	835,860	17,354	16,664	312,655	14,150
	Cluster Factor	1.01	1.08	1.03	1.03	1.09	1.06
	Endogenous DNA (%) Post-Dedup	0.99	6.41	0.11	0.13	2.13	0.09
	5’ C>T 1st base (%)	17.40	15.80	10.64	10.96	13.87	13.89
	5’ C>T 2nd base (%)	1.90	1.07	1.73	1.31	1.51	1.55
	3’ G>A 1st base (%)	20.60	16.61	11.91	11.08	14.22	11.85
	3’ G>A 2nd base (%)	1.30	1.17	1.75	1.63	1.61	2.21
	Mean read length (bp)	46.73	53.40	48.13	49.11	49.10	47.75
	Median read length (bp)	44	49	43	44	46	43
**Sex DetERRmine**	Rate X	0.79	0.45	0.66	0.70	0.88	0.59
	Rate Y	0.00	0.30	0.08	0.11	0.04	0.46
	Err Rate X	0.0560	0.0039	0.0357	0.0377	0.0096	0.0380
	Err Rate Y	0.0000	0.0105	0.0407	0.0477	0.0064	0.1096
	**Sex Assign**	**F**	**M**	**U**	**U**	**F**	**M**
**Rx ratios**	Rx	0.84	0.49	0.71	0.69	0.94	0.62
	95% CI	0.78–0.89	0.47–0.51	0.67–0.76	0.66–0.73	0.89–0.98	0.59–0.66
	**Sex Assign**	**F**	**M**	**U**	**U**	**F**	**M**
**Calculation of the X chromosome read dosage** **Gower et al. (2019)**	Read dosage on the X chromosome (Mx)	0.83	0.49	0.70	0.689	0.93	0.62
	Reads mapped to the X chromosome (Nx)	349	20,909	629	591	14,649	450
	Reads mapped to the autosomes (Na)	7,980	809,705	16,622	15,957	29,6732	13,606
	**Sex Assign**	**F**	**M**	**U**	**U**	**F**	**U**
**mtDNA**	Number of reads mapping to the rCRS	7,143	8,828	-	-	4,984	-
	Haplogroup	**H3ap**	**HV24**	-	-	**J1b1a1**	-
**Y-chromosome**	Haplogroup	**-**	**J1**	-	-	**-**	-

The proportion of endogenous DNA estimated for this set of samples varied between 0.09% - 6.41% for the shotgun sequencing data. Only three individuals (1092-F, 1647-F and 955-H) passed quality thresholds to undergo genetic analysis due to poor DNA preservation. Mitochondrial DNA (mtDNA) haplogroups were identified for the individuals determined to be female, HS-1584 (H3ap) and HS-931 (J1b1a1), and the male 2334-F (HV24). In the latter case, we also identified the Y-chromosome haplogroup J1. The mtDNA haplogroups provide information about the maternal lineages while Y-chromosome haplogroups reflect paternal lineage. The mtDNA haplogroup H3 is found throughout Europe and in the Maghreb and has been found in Neolithic sites in Portugal. Its particular prevalence in populations in Andalusia and Morocco is thought to be a result of prehistoric migrations between these regions across the Straits of Gibraltar [134–136]. It has a postglacial or early Holocene origin, ~9500 ya in Iberia and ~8100 ya in North Africa, probably as a consequence of old introgression from Iberia to North Africa coinciding with largescale population resettlement within Europe [137]. MtDNA haplogroup J1b1a1 has an estimated Neolithic age of 9175 ± 3092 years and is widespread in present-day Near East, North Africa, the Caucasus and northern and central Europe, having likely spread during postglacial migrations [138, 139]. Concerning the male individual, 2334-F, the mtDNA haplogroup HV (excluding subclades H and V) is not particularly common among present-day Europeans (0–10%), being extremely rare in Iberia and reaching the highest frequencies in southern Italy. This haplogroup is also found at high frequencies in Iran and the Middle East [140]. The same individual harbours the Y-chromosome haplogroup J1, which has distinctly Middle Eastern origins, and a high incidence in North Africa. Specifically, this haplogroup reaches ~40–80% frequency across the Arabian Peninsula and ≥ 30% in Tunisia, Algeria and Morocco. In contrast, it has an average frequency of ~5% in Europe and <10% in Iberia.

### 6.7.) Reconstruction of solar position results

Using the online model SunCalc [121], it could be observed that in January, or midwinter, the sun rises to the east south-east of Santarém, and could be used to estimate the direction of Mecca quite accurately. In June, or midsummer, the sun rises to the north-east of Santarém but sets to the north-west, so if 180° was calculated from the setting sun in summer, a relatively accurate estimation for the direction of Mecca could also be achieved. In Spring and Autumn, however, the sun rises approximately in the east and sets approximately in the west of Santarém which would reduce the accuracy of predicting the direction of Mecca, regardless of whether the sunrise or sunset was used.

## 7.) Discussion

### 7.1) Grave orientation

#### 7.1.1.) Grave orientation: Patterns

**Fig 10:**
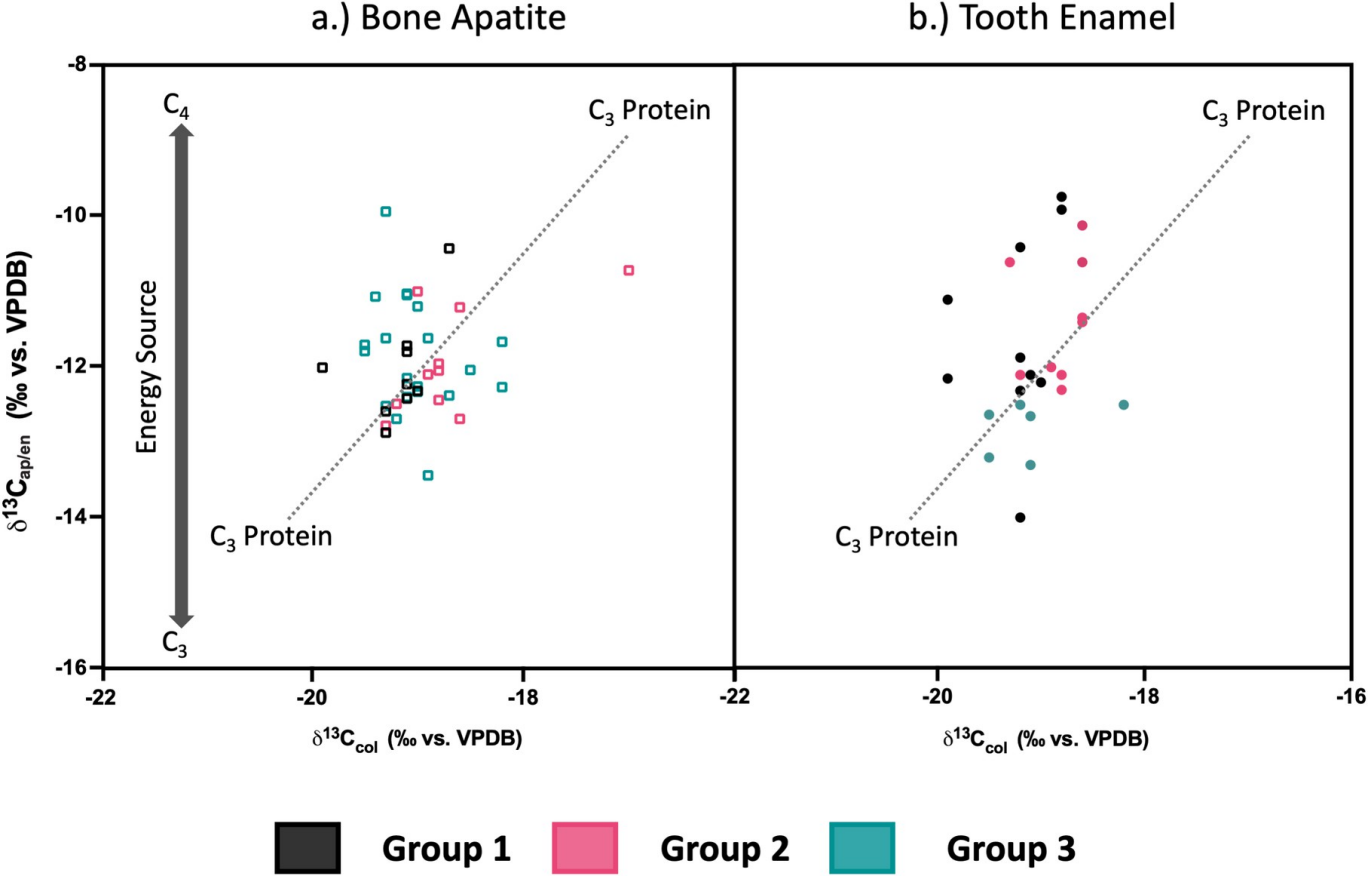
Human (a.) bone collagen (*δ*^13^C_col_) vs bone apatite (δ^13^C_ap_) and (b.) bone collagen (*δ*^13^C_col_) vs tooth enamel (δ^13^C_en_), by grave orientation (plotted against C_3_ protein regression line [131] indicating main protein and energy sources in diet).

**Fig 11:**
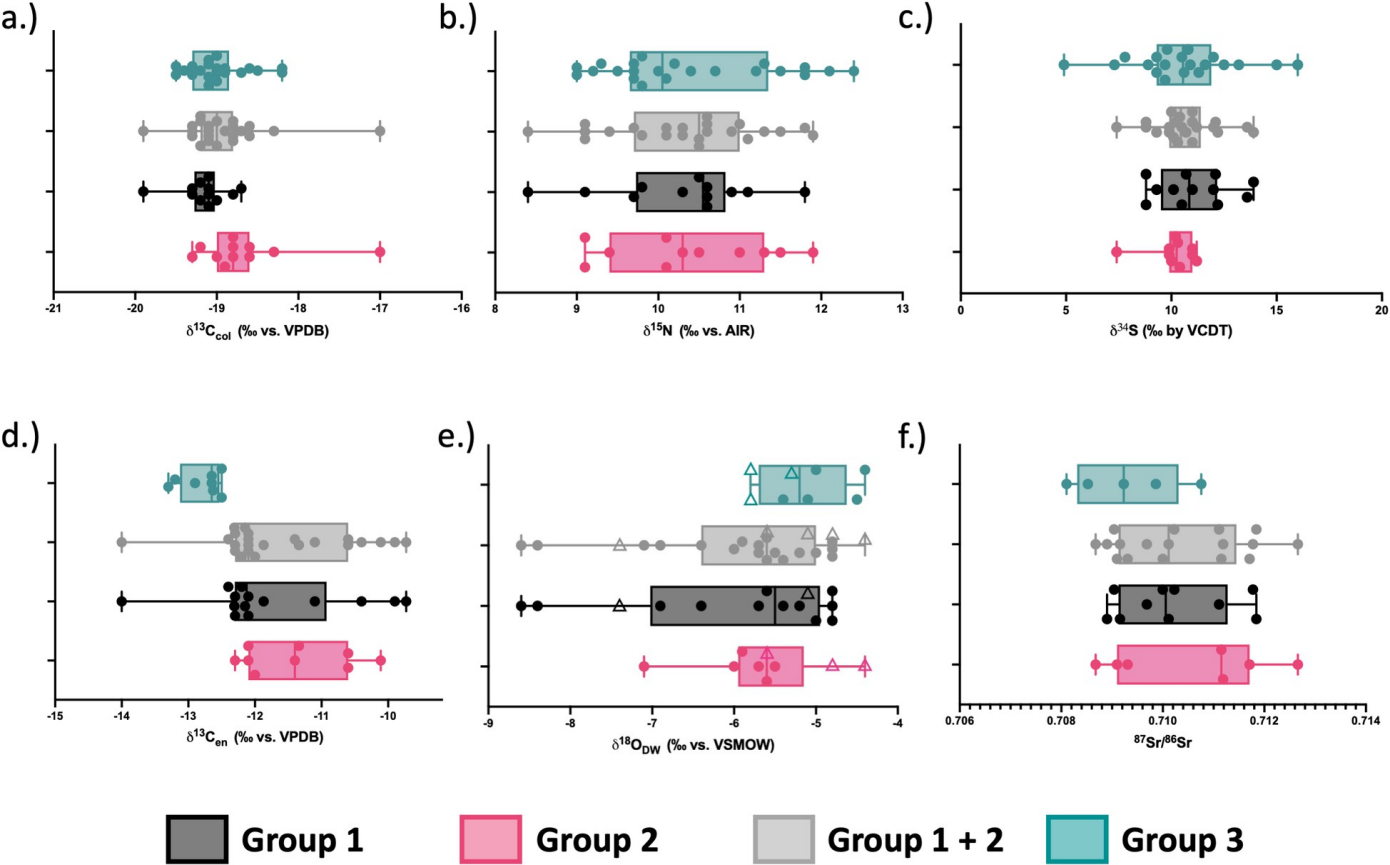
Isotope results according to grave orientation. a.)*δ*^13^C_col_, b.) *δ*^15^N, c.) *δ*^34^S, d.) δ^13^C_en_, e.) *δ*^18^O_DW_ and f.) ^87^Sr/^86^Sr. M1 teeth are indicated by triangles in 9e.

Medieval Islamic burials are typically sparse by nature. While it is generally accepted that the Muslim conquest of Iberia brought with it a largely Berber population along with an Arab ruling elite class [76, 141], the paucity of grave goods leaves no indication of wealth or social status for those buried at the time. Indeed, it is only in exceptional cases that status can be inferred from medieval Islamic burials at all, for example in São Jorge Castle, Lisbon, where the location of burial was strong evidence of importance [20]. Although it is not possible to distinguish the status of the individuals from Avenida 5 de Outubro, the varying orientations of the graves themselves provides a different parameter within which to consider the above data. As explained in section 4.1.1., the individuals were grouped according to grave/skeleton orientation for the purpose of discussing the data. It should be noted, however, that although three distinct groups could be observed, it is not clear if the unorthodox burials (i.e. Group 1 and Group 2) are meaningfully separate from each other, or just differ as a whole from the canonical burials (Group 3). To this end, results are discussed with consideration to each of the groups and are also represented accordingly in the Figs 10 and 11, but in the latter figure, Group 1 and Group 2 are also represented as a potential combined group of unorthodox burials (Group 1 + 2).

Considering the carbon isotope data, at first look (see Fig 7) it appears that the whole diet inputs for this population in both childhood and adulthood are similar mixes of mainly C_3_ with some C_4_ inputs, but when the results are framed in terms of the grave orientation of the individuals, a clearer picture emerges. When the *δ*^13^C_col_ and *δ*^13^C_ap_ is compared, there are no clear differences between the individuals with Group 1, 2 and 3 graves, as seen in Fig 10A. However, when the *δ*^13^C_en_ is compared to the *δ*^13^C_col_ in Fig 10B, the range of *δ*^13^C_en_ values is wider for the ‘Group 1’ individuals, over 4‰, than for the ‘Group 3’ individuals at 0.8‰. A Kruskall-Wallis H test identified a significant difference between the three groups in the mean ranks of the *δ*^13^C_en_ values (H = 10.795; p = 0.0045) and a Post-hoc Dunn’s test with a Bonferroni correction identified a significant difference between the following pairs: Group1 + Group3 and Group2 + Group3. A significant difference was also found in the mean ranks of the *δ*^13^C_col_ values (H = 6.365; p = 0.0415; Post-hoc Dunn’s test with a Bonferroni correction: Group 1 + Group 2) but considering that all the *δ*^13^C_col_ values except for HS-921 reflect typical C_3_ plant consumption, this latter difference is not thought to be meaningful in a dietary sense (see S3 Text) [122]. With the exception of one individual from Group 1 (2334-F M1: -14‰), all Group 1 and Group 2 individuals had *δ*^13^C_en_ values that were more ^13^C-enriched than Group 3, indicating a higher proportion of C_4_ plant input in childhood diet, or at least had more varied diets than the individuals who had Group 3 graves, a pattern that seems to disappear in adulthood based on the bone *δ*^13^C_ap_ values for all groups. In fact, 2334-F also had a later formed tooth sampled (M3) that was also more ^13^C-enriched (-11.9‰). Due to its high nutritional value, in many African cultures millet porridge is eaten by pregnant and lactating women and children [85]. While millet was consumed in Europe prior to the Muslim conquest, it is possible that the higher *δ*^13^C_en_ values of Groups 1 and 2 reflect either a food source preference or cultural practice, of consuming millet or sorghum porridge perhaps, that could be an indicator of individuals spending their childhoods in regions where this practice was more common or that the tradition persisted among some of the Muslims living in Santarém. In contrast, in Group 3, individuals consumed less to no C_4_ plants in early childhood.

The *δ*^18^O values in the tooth enamel also display a distinct pattern when considered in terms of grave orientation of the individuals. While the *δ*^18^O_DW_ values of the Group 3 individuals all fall between -4.4‰ and -5.8‰, which is consistent with modern precipitation in the region around Santarém, paleoclimatic reconstructions have indicated that during the climatic periods of the Dark Ages (DA: 500–900 AD) and the Medieval Climate Anomaly (MCA: 900–1300 AD), conditions were warmer and drier over the southern Iberian Peninsula, resulting from a positive North Atlantic Oscillation (NAO) phase [74, 75, 142, 143]. Given the regional aridity during this time, one could expect less negative *δ*^18^O_DW_ values in individuals who spent their childhood in Santarém or nearby. Some of the teeth (1/9) sampled from Group 2 and 5/14 from Group 1 reflect *δ*^18^O_DW_ values that are more negative than -6‰, illustrated in Fig 11E and one female individual (HS 1442) had values as low as -8.4‰ in her M2 and -8.6‰ in her M3. These values indicate possible origins in a region with more humid conditions than Santarém at this time but could be consistent with precipitation *δ*^18^O values in the wetter mountainous regions of the Maghreb [53, 54, 69, 70]. Origins in the Arabian Peninsula seem less likely, as it is very arid and less negative *δ*^18^O_DW_ values may be expected. It should also be considered that precipitation *δ*^18^O values in the less humid areas of the Maghreb also overlap with expected *δ*^18^O values around Santarém [69], so it is not only the individuals with the most negative *δ*^18^O_DW_ values that may have migrated from North Africa, but it is not possible to distinguish the others using only oxygen isotopes as a proxy.

Overall, the relative homogeneity of Group 3 in both the *δ*^18^O_DW_ values and the *δ*^13^C_en_ values in their teeth seems to indicate similarity in their drinking water sources and in whole diet plant inputs, which would be consistent with a local population in Santarém who sourced water locally and ate a predominantly C_3_-based diet in childhood. In contrast, the Group 1 and Group 2 individuals display more heterogeneity overall (see Fig 11D). While this pattern did not prove to be significant statistically in the *δ*^18^O_DW_ values (see S3 Text) [122], it is still present practically, with a min-max difference of 4.2‰ in Group 1+2 compared to 1.4‰ in Group 3. The large geographic variability for precipitation *δ*^18^O_DW_ values, along with the considerable overlap across the regions of interest, undoubtedly makes it very difficult to distinguish which individuals may have been non-local. These isotope values can be interpreted as a general pattern of mobility and behavioural differences amongst the individuals buried with different orientations i.e., some of the Group 1 and Group 2 individuals moved from a more humid region, possibly the wetter parts of the Maghreb, to Santarém, and both of these groups consumed more C_4_ plants in childhood, perhaps sorghum or millet porridge, as is still a popular staple food throughout North Africa. If these individuals were indeed Berber settlers, they may have migrated to Iberia from different parts of North Africa, which would explain the more heterogenous isotope values of these two groups compared to the relatively homogenous Group 3.

Regarding the aDNA, it is important to consider that the North African gene pool has been formed by Palaeolithic and Neolithic back-migration of several European lineages, with Berber populations in particular being very genetically heterogenous [135, 144, 145]. The mtDNA haplogroups identified in three individuals from this population are found in Europe but also relatively frequent in the Maghreb. In fact, the H3 and HV haplogroups, which are found in mean frequencies of around 18% and 5%, respectively, in Mediterranean Europe, are within the same range in the Maghreb (13% and 7%, respectively). These three individuals belonged to Group 1 (2334-F and HS-931) and Group 2 (HS-1584) and their maternal lineages do not exclude Berber origins, in fact when combined with the overall previously mentioned patterns of data for these groups, it seems even more likely. Taking into account the clear difference in the M1 *δ*^13^C_en_ value for 2334-F, compared to the other Group 1 and Group 2 individuals, as well as his apparent Middle Eastern genetic lineage (see section 5.6), it is plausible that his tooth enamel reflects early childhood dietary practices that were more influenced by Arab culture, in which many of the most popular dishes at the time made use of wheat and/or barley e.g. *tharīd*, a bread stew [146], rather than North African Berber influences which may have included higher consumption of C_4_ plants. It is clear though, that C_4_ plant consumption by this individual increased during the time frame of M3 enamel formation.

The ^87^Sr/^86^Sr ratios of this population showed an overall consistency with the bioavailable ^87^Sr/^86^Sr range, as previously mentioned, with the exception of some individuals who had ^87^Sr/^86^Sr ratios below 0.709. When considered by grave orientation, there are no clear differences between the groups (see Fig 11F). It should be noted, however, that the consistency of human ^87^Sr/^86^Sr ratios with measured bioavailable ranges does not serve as absolute confirmation of a local origin, but only as evidence of provenance in a geologically similar region. In fact, ^87^Sr/^86^Sr ratios measured in studies across the Maghreb have also yielded similar ranges, for example 0.710–0.712 in human tooth enamel from southwestern Libya [147], 0.708–0.710 in plant ash from northern Tunisia [70] 0.7078–0.7096 in waters from the Continental Intercalaire (CI) aquifer that flows from the Atlas Mountains in Algeria to the Tunisian Chotts [148] and 0.7093–0.7105 in groundwater from the Lakhssas Plateau in Morocco [149]. This means that Berber populations who migrated from the Maghreb and settled in Santarém would not necessarily have very distinct ^87^Sr/^86^Sr ratios from individuals who had spent their entire lives in the vicinity of Santarém.

**Fig 12:**
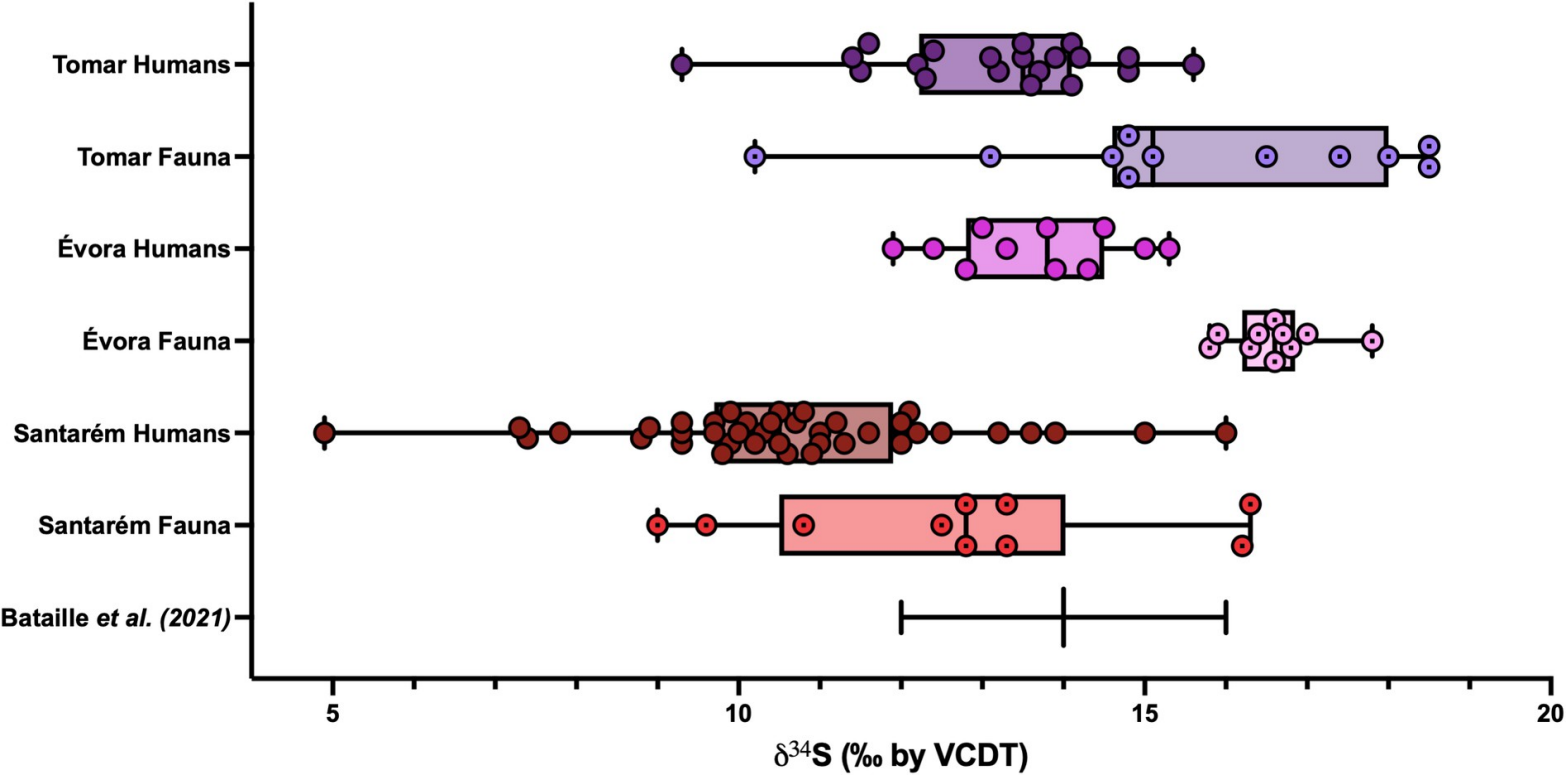
Human and fauna *δ*^34^S from Santarém (this study), Évora [22] and Tomar[19] along with expected regional range [47].

Another potential indicator of mobility is sulphur. Fig 12 compares the Santarém human and fauna *δ*^34^S results to the data from the sites of Tomar (11^th^-17^th^ C) [19] and Évora (12^th^-13^th^ C) [22] along with the predicted range [47] and demonstrates that the Santarém population is highly variable in comparison to both Tomar and Évora. With the exception of two ovicaprids (FVRP3; FVRP9) and the fox (FVRP5), the fauna fell within the predicted range while many of the humans had *δ*^34^S values below 12‰. It is plausible that the human *δ*^34^S values are highly variable because the time during which the individuals lived (and when the *δ*^34^S was incorporated into their bones) was during the period in which high Saharan aeolian input deposited sulphur from northern Africa in Iberia [143]. This potentially would have caused fluctuating atmospheric and soil *δ*^34^S but the extent of this influence is unclear

The *δ*^34^S values, were the most heterogenous in the Group 3 individuals (ranging from 4.9‰ to 16‰), while the Group 1 (ranging from 8.8‰ to 13.9‰) and Group 2 individuals had more homogenous *δ*^34^S values (ranging from 7.4‰ to 11.2‰) (see Fig 11C). This pattern is in contrast to the *δ*^18^O_DW_ and *δ*^13^C_en_ data, but when Group 1 and Group 2 are considered together, this pattern mostly disappears, and the range is similar to that of Group 3. It is important to reiterate that these *δ*^34^S values were measured in the bone collagen and reflect sulphur incorporated into the bones over a period of years before the death of the individual while the tooth enamel is formed over a much shorter space of time and a much earlier period of the individual’s lives. It is possible that diverse dietary input in the years prior to death might also explain such high variability and the fact that some human *δ*^34^S values are lower than the modern expected range for Santarém. If this is indeed a dietary pattern, it is not apparent in the *δ*^13^C_col_ and *δ*^15^N values, adding an additional layer of complexity to the interpretation of this data.

While *δ*^34^S may not have provided clarification regarding the mobility of the Santarém population, the *δ*^34^S values obtained are still an important contribution in a broader sense because there is so little published data for medieval Portugal, and no studies (to date) have provided *δ*^34^S values for a human population living during the Late DA (650–900 AD) and during the Early MCA (900–1100 AD) in Portugal. This was a notable climatic event, so making *δ*^34^S data available for this region during this time period may prove useful to future studies.

#### 7.1.2.) Grave orientation: Temporal relationships

Taking into account the estimation of the position of the sun in various seasons, it is plausible that many of the Group 1 individuals may have been buried during the Spring or Autumn, facing towards the sunrise and thus facing E instead of towards Mecca. The Group 2 individuals could have been buried closer to midsummer or midwinter, resulting in slightly more accurate positioning but still not well-aligned to Mecca. It cannot be ruled out that at least some of the well-aligned Group 3 burials may have been buried in midsummer or midwinter when the direction of Mecca could be most accurately estimated based on sunset or sunrise, respectively. The isotope data from the tooth enamel of the Group 3 individuals indicate a pattern of homogeneity in both the *δ*^18^O_DW_ and *δ*^13^C_en_ values, compared to the heterogenous values of the Group 1 individuals, which could be explained by a difference in childhood dietary habits (for *δ*^13^C_en_) and regional drinking water sources (*δ*^18^O_DW_) as previously discussed. This pattern is unlikely to be accounted for, however, if the differences in grave orientation were only a result of the individuals being buried in different seasons. While the initial archaeological interpretation of the site introduced the idea of a linear evolution from ‘poorly-oriented’/unorthodox to ‘well-oriented’/canonical burials that were more likely to be deposited after the *aljama* mosque was built and the correct orientation of graves was standardized [91, 93], the reality is probably much more nuanced. The reappraisal of the stratigraphy data suggests that in fact the Group 3 graves might be older than some of Group 1 and 2 graves. Although some Group 3 graves might be older, we cannot at this moment say with any certainty that all individuals buried the same way are chronologically contemporaneous. If some Group 3 graves were more recent, as the initial interpretation of the site had suggested, [91, 93] then the data pattern would support the presence of a more mobile population, with childhood dietary inputs of sorghum and millet (a possible Berber/North African cultural influence) in the earlier stages of the Islamic conquest (Groups 1 and 2) as well as a more sedentary, C_3_ plant consuming population (Group 3) at a later stage in Santarém.

Alternatively, if the Group 3 burials correspond to the first stage of use of the necropolis, they may reflect a population of local people, converted to Islam. Although the Umayyads did not generally force conversions in the extant Christian inhabitants of Iberia [79, 80], they may have occurred as a result of interfaith marriages, which were frequent in the early decades of the conquest of Iberia, and were important in the process of consolidating territory and power [150]. The unorthodox burials of Group1 and Group 2 may therefore correspond to a heterogenous population (or populations) arriving in Santarém at a later phase. They may, in fact, be related to the Late Caliphate efforts of ‘islamisation’ and reinforcement of control over this peripheral territory, which became a frontier after Christian Kingdoms took Coimbra in the late 9^th^ century. Although the written sources provide very little information regarding this episode, the construction of a mosque in Santarém [92], renovation work on the walls in Lisbon in 985 AD [151] as well as changes in pottery styles in the 9^th^-10^th^ centuries all point towards a dominance and triumph of Islamic traditions [152]. The efforts to reinforce and consolidate the caliphal dominion is likely to have introduced waves of foreigners from elsewhere, including North Africa, either through military campaigns or due to the attractive nature of a dynamic city. What matters more than the chronological relationship of varying orientations in this necropolis, is the fact that the data supports a difference between those in unorthodox burials and those in canonical burials. The pattern of data for Group 1 and Group 2 would support the influx of waves of non-locals to Santarém and although the chronology is not (at this stage) very certain, we still see the clear evidence of human movement, through this study, that is not well documented in historic sources.

### 7.2.) Sex

**Fig 13:**
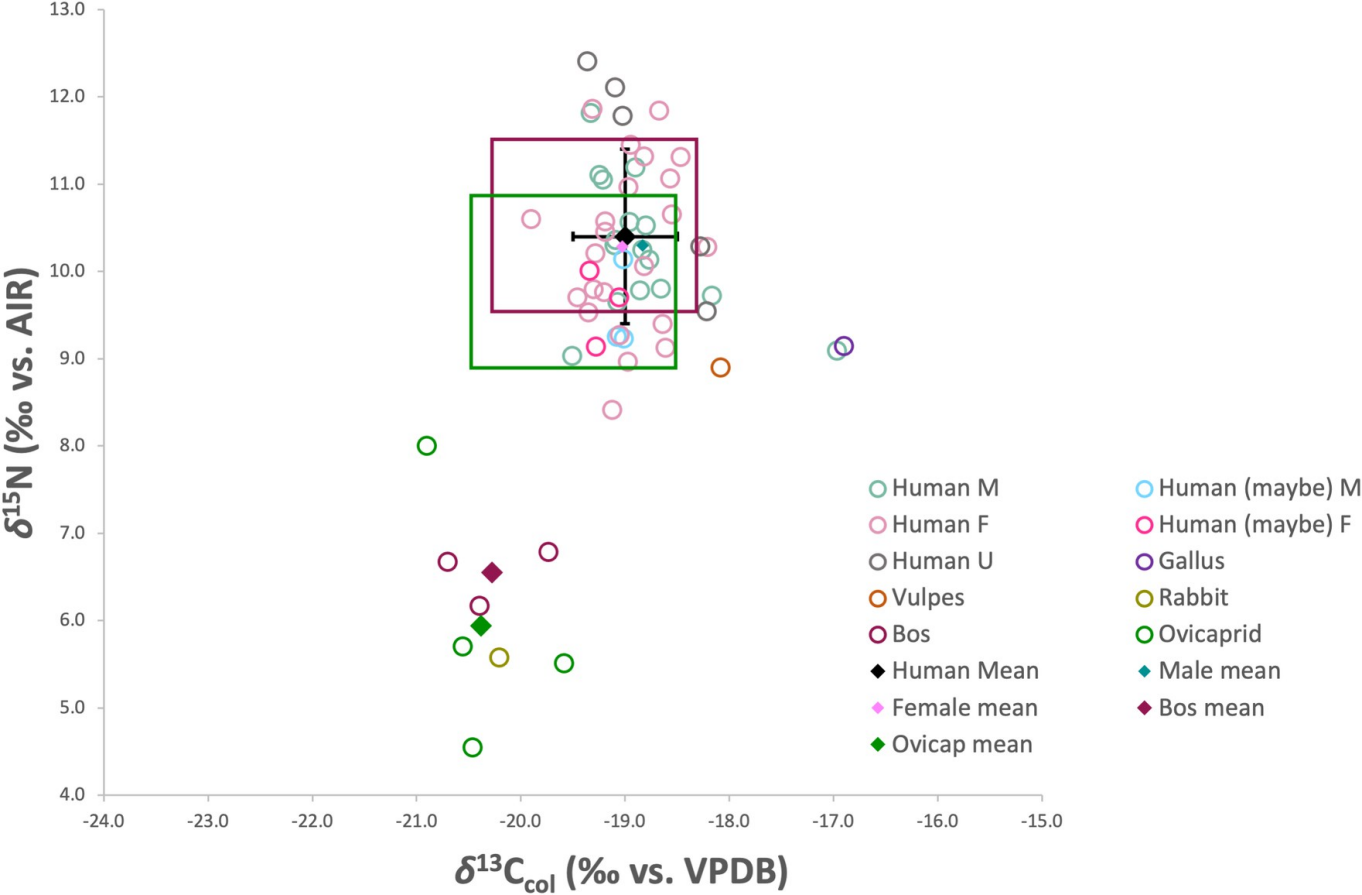
Human and fauna *δ*^15^N and *δ*^13^C_col_ by sex. Squares represent expected range of +1 trophic level increase (+0–2‰ in *δ*^13^C_col_; +3–5‰ in *δ*^15^N) over mean *Bos* and Ovicaprid values, respectively.

Excluding the results of the skeletons classified as ‘Maybe F’, ‘Maybe M’ and ‘U’, the data was assessed for patterns in terms of sex. There were no clear differences in the *δ*^13^C_col_ and *δ*^15^N according to sex, with males (n = 14) having a mean value of -18.8 ± 0.6‰ in *δ*^13^C_col_ and 10.3 ± 0.8‰ in *δ*^15^N, while females (n = 20) had values of -19.0 ± 0.4‰ and 10.3 ± 1.0‰ for *δ*^13^C_col_ and *δ*^15^N, respectively (Fig 13). The *δ*^34^S values for males (n = 10) were slightly higher (*δ*^34^S = 11.9 ± 2.1‰) than females (n = 18; *δ*^34^S = 10.7 ± 1.7‰) but were not significantly different. This indicates that adulthood diet was relatively similar amongst males and females in the Early Islamic Period in Santarém.

**Fig 14:**
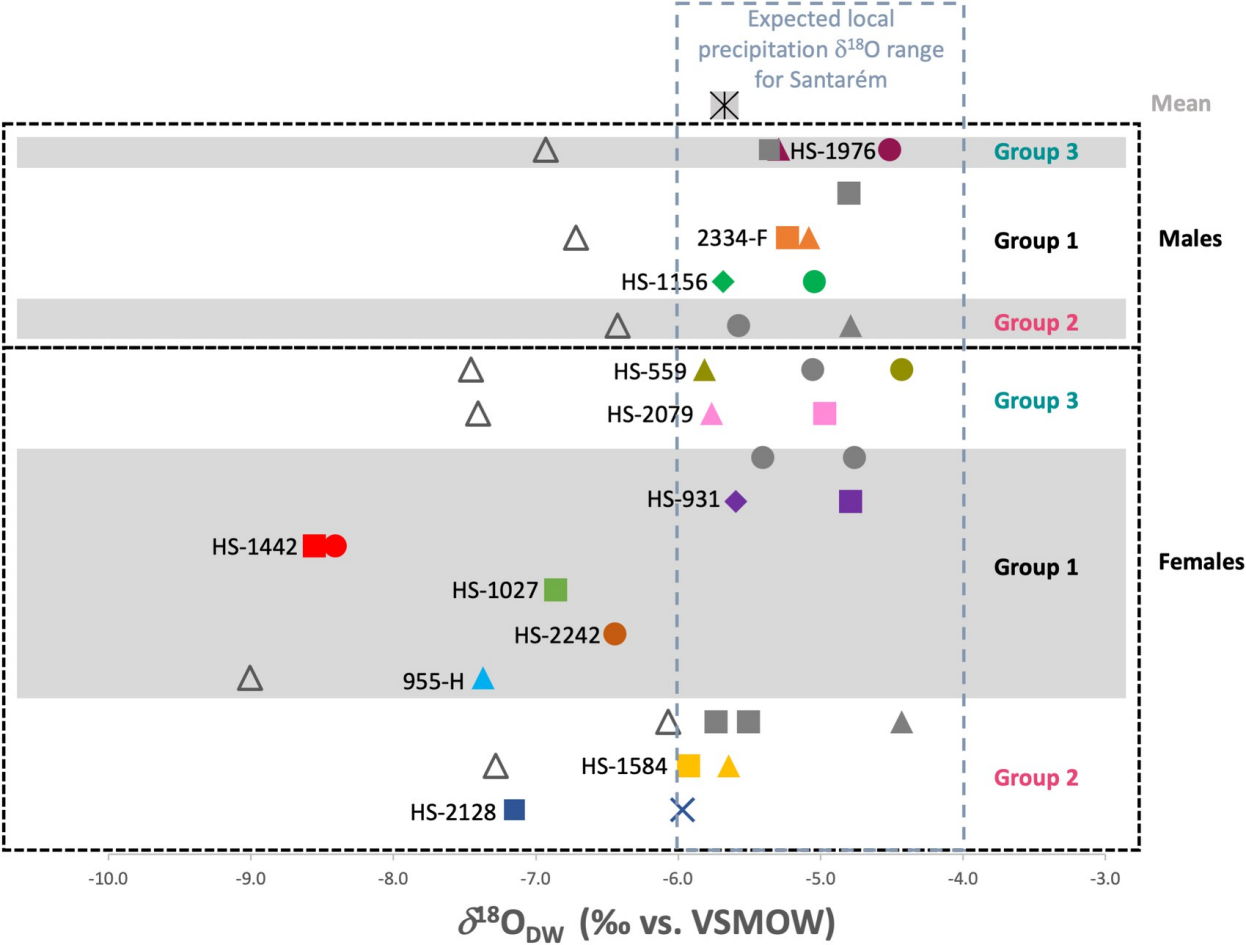
Human *δ*^18^O_DW_ with multi-tooth comparison. Individuals with values outside of local range and/or of whom multiple teeth were sampled are in colour and labeled. Individuals within local range and with only one tooth sampled are in grey. Solid triangles = M1s, circles = M2s, squares = M3s, cross = PM1 and diamond = PM2. Hollow triangles represent hypothetical M1 values corrected (-1‰ from *δ*^18^O_c_ VPDB) for breastfeeding.

Considering the tooth enamel results for *δ*^13^C_en_ and *δ*^18^O_DW_, the males (n of teeth = 10, from 7 individuals) have slightly more variable carbon (*δ*^13^C_en_ = -12.0 ± 1.3‰) than the females (n of teeth = 21, from 15 individuals; *δ*^13^C_en_ = -11.9 ± 0.8‰) while the females show some more variability in their oxygen (*δ*^18^O_DW_ = -5.9 ± 1.2‰) than the males (*δ*^18^O_DW_ = -5.1 ± 0.4‰). While it is true that the sample size of teeth was larger for females than for males, the number of individuals with teeth available for sampling was proportionally low and more females had teeth available for sampling than males. Although we cannot assume that this small number of samples is fully representative of the whole Muslim population under study, it is nevertheless interesting that all of the *δ*^18^O_DW_ values below -6‰, i.e., possibly reflecting origins in a more humid region than Santarém, belong to five female individuals (1442-F, 955-H, HS-2242, HS-1027, HS-2128). The last belongs to Group 2 while the other four were in Group 1 graves. The M1s, for which *δ*^18^O_c_ values were corrected by -1‰ and the *δ*^18^O_DW_ values recalculated, are represented by the hollow triangles in Fig 14, which demonstrates the distribution of *δ*^18^O_DW_ values in terms of both sex and grave orientation. Although it is just a conservative estimation [132, 133], if this correction of -1‰ were a true reflection of breastfeeding enrichment in the M1s, it would mean that most of the individuals have *δ*^18^O_DW_ values out of the expected precipitation range for Santarém, with the most negative values belonging to the females.

**Fig 15:**
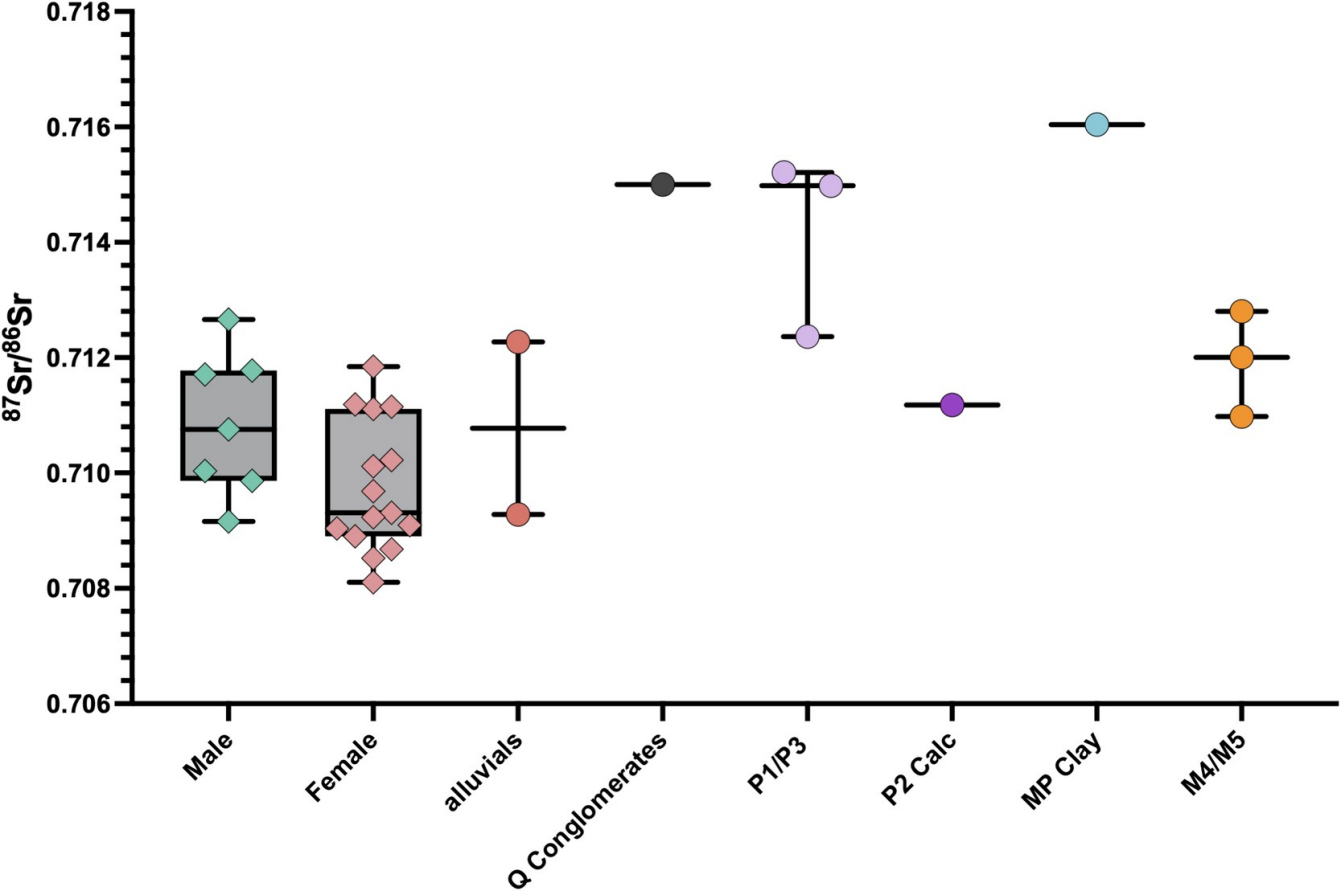
Human ^87^Sr^/86^Sr results by sex compared to bioavailable ^87^Sr^/86^Sr for Santarém (plant ash samples from this study and James *et al*. 2022 [61]).

**Fig 16:**
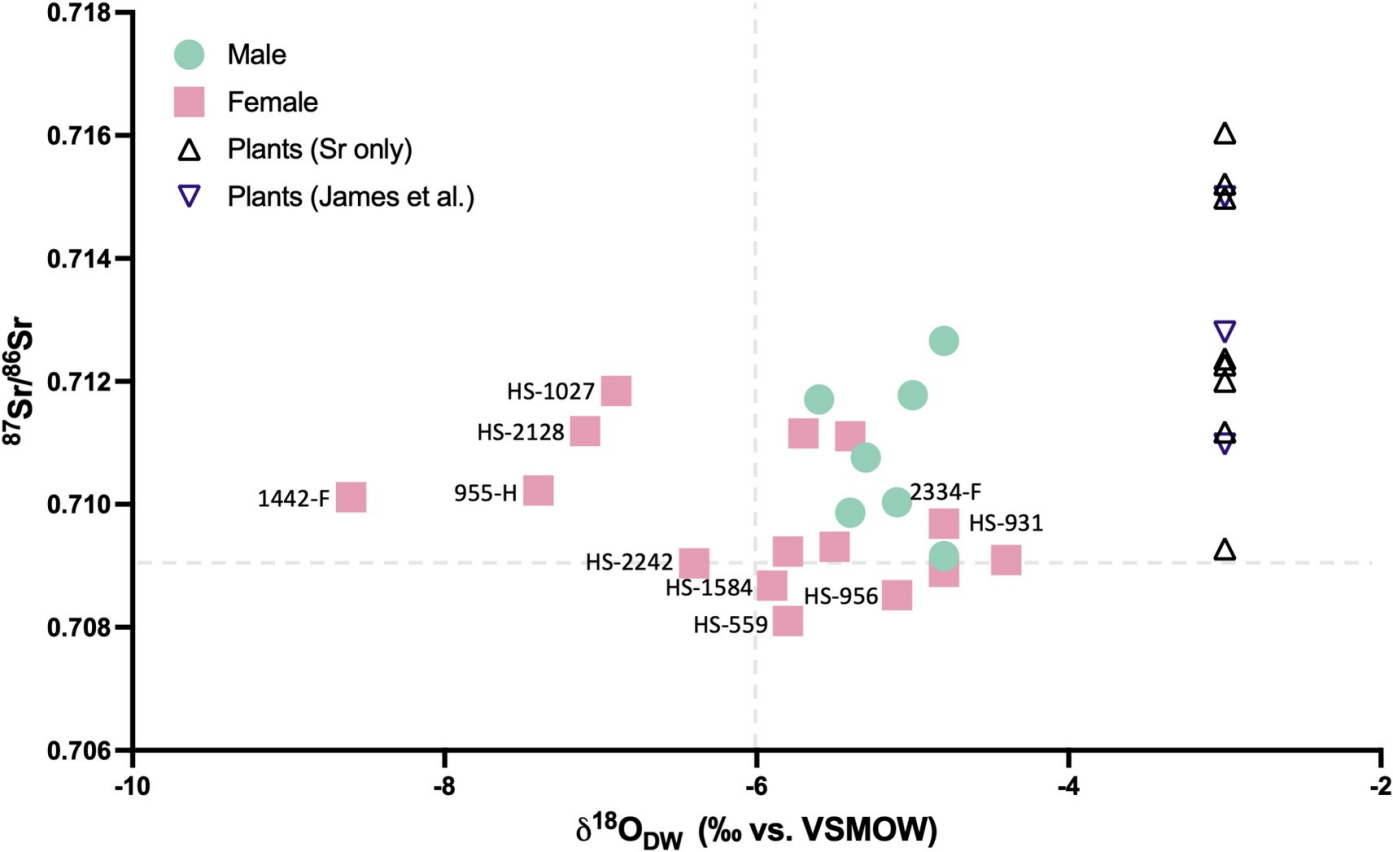
Human ^87^Sr^/86^Sr and *δ*^18^O_DW_ results by sex. Grey lines indicate the limit of the expected ‘local’ range for the respective isotopes in Santarém. For ^87^Sr^/86^Sr, bioavailable range is determined from plant ash samples from this study and James *et al*. 2022 [61].

Taking the ^87^Sr/^86^Sr ratios into account too, the females were also slightly more variable than the males. Bioavailable ^87^Sr/^86^Sr ratios below 0.709 were not obtained for any of the geological substrates surrounding Santarém (see Fig 15) but the three individuals who had tooth enamel ^87^Sr/^86^Sr ratios below 0.709 were all female. Although this difference may appear small, it would also be consistent with examples of the ranges of ^87^Sr/^86^Sr in North Africa that were previously discussed. While tentative, the consideration of *δ*^18^O_DW_ and ^87^Sr/^86^Sr together, as demonstrated in Fig 16, would indicate a pattern of more sedentary males and more widespread origins in the females. Although it is necessary to interpret this data with caution, due to the small number of tooth enamel samples, particularly for the males, it could support the argument for a patrilocal social system whereby the males were more sedentary, and females relocated later in life, possibly upon marriage.

Furthermore, three of the female individuals (955-H, HS-931 and HS-1584) had cranial morphometric features, such as maxillofacial prognathism, broad nasal apertures, smooth inferior nasal margins, retracted chins and rectangular occipital orbits, that are more prevalent in pre-modern populations with African genetic ancestry than Eurasians [102, 103]. While it is true that these characteristics could be retained in the Muslim population for many generations after migrating to Iberia, the *δ*^18^O_DW_ value in the M1 of 955-H suggests that at least one of these individuals may have spent their childhood in a different region, hence could have been a first generation migrant to Santarém. The mtDNA results for HS-931 and HS-1584 also support maternal lineages that are frequent in the Maghreb and Arabian Peninsula (haplogroups H3 and J1b respectively). Genetic diversity is higher amongst females than males in Berber populations, due to patrilocality [144, 153], so if this practice continued in the Santarém population it could explain the previously mentioned isotope data patterns for the females as a whole.

The male individual, 2334-F, appears to have Arabian genetic origins, certainly in paternal lineage and probably maternal lineage too. Genetic studies on modern populations in the Maghreb found that J1 (Arab) lineages comprise 17% of the modern male Tunisian gene pool and the Arab male genetic input from 7^th^ century expansions is thought to be as high as 38% in Tunisia, and also high in Algeria [144, 153]. Considering that the Arabisation of the Maghreb, and the Islamic conquest of Iberia were military ventures, most likely driven by Arab men displacing native Berber men but marrying Berber women, in the case of the former, it makes sense to have females with apparent European/Berber genetic lineages and a male with Arab genetic lineage. As discussed in section 7.1.1, the early childhood diet of this male, 2334-F, is also distinct from the other Group 1 and Group 2 individuals, which could be a consequence of different food practices related to cultural influences (Arab/Berber) if his genetic lineage is a clue to his lived experience. It is impossible to speculate broadly based on three individuals, but the isotope results of the rest of the Santarém population has also been informative. There are records of Muslim men marrying local non-Muslim women who subsequently converted in the years following the conquest of Iberia, for example the widow of King Roderic, Egilona, who married the Berber Abd al-Aziz, son of the conquering general Musa bin Nusayr [154]. This was likely to have been a relatively common practice as it consolidated Muslim authority, ensured legitimacy over inherited lands, and was a vital element for driving the process of social and cultural change in Iberia following the conquest [150, 154]. While this may indeed have been the case for some of the females from Santarém, whose *δ*^18^O_DW_ values and ^87^Sr/^86^Sr ratios are consistent with expected local ranges, there are at least some (previously mentioned) females who appear to have migrated from a region with higher precipitation and/or possibly lower ^87^Sr/^86^Sr ranges, in the cases of HS-1584, HS-559 and HS-956, for example the Atlas Mountains region of Morocco and Algeria.

## 8.) Conclusion

The characteristic simplicity of medieval Islamic graves, combined with the relative paucity of historical documentation concerning the first centuries following the Islamic conquest of Portugal and Spain, has historically presented an obstacle to detailed archaeological interpretations of the lives of populations settling in the Iberian Peninsula during this period. The multi-isotopic analysis of the skeletal material from Avenida 5 de Outubro #2–8 has provided a unique opportunity to better understand the dietary habits and mobility of this early Muslim population in Santarém, Portugal. Taking into account one of the few distinguishing characteristics among the burials, the orientation of the graves and skeletons, and using *δ*^13^C_col_ and *δ*^15^N analysis, it was possible to identify a pattern of higher consumption of C_4_ plants during childhood in the individuals ‘incorrectly’ aligned towards Mecca compared to those in ‘correctly’ aligned graves, whilst the adulthood diet was somewhat homogenous in terms of consumption of mainly C_3_ plants with some C_4_ input and local C_3_-fed domestic herbivores. Stable oxygen isotope analysis revealed a mobility pattern of relative homogeneity amongst the individuals in canonical/‘correctly’ aligned graves, consistent with expected regional precipitation values, while the unorthodox/‘incorrectly’ aligned individuals were more variable and some likely spent their childhood in more humid regions with higher precipitation than Santarém (for example, the wetter mountainous regions of the Maghreb). These individuals may have belonged to different waves of foreign settlers arriving in Santerém in the case of groups 1 and 2, while group 3 likely comprises of local individuals.

It was also possible to observe that many of the individuals with apparent non-local *δ*^18^O_DW_ values were female. While this pattern may have some bias due to available sample size, it appears to suggest a possible patrilocal system of social behaviour with a high degree of mobility amongst females after childhood. These aspects of dietary and social behaviour have not been visible in the historical record until now, so this study has served to confirm the value of multi-isotopic analysis as a means to confirm or support archaeological interpretations, and provide additional detail to complex historical settings.

## Supporting information

S1 Table δ^13^C and δ^15^N uncertainties calculated from Szpak et al. (2017).
(XLSX)

S1 Text Ancient DNA analysis. (DOCX)

S2 Text Bone apatite preservation. (DOCX)

S3 Text Statistical comparison of results. (DOCX)
