# Supplementary material for: Shrouded in history: Unveiling the ways of life of an early Muslim population in Santarém, Portugal (8th– 10th century AD)
Source: PLoS One. 2024 Mar 6;19(3):e0299958. doi: 10.1371/journal.pone.0299958 (PMC10917335; doi:10.1371/journal.pone.0299958)
Supplement: S1 Text — (DOCX) [file pone.0299958.s002.docx]

**Supporting Information (S2 Text)**

**S2. Ancient DNA analysis**

The raw sequencing reads were processed, analysed and mapped against the human reference genome hg19 using the *nf-core/eager* bioinformatic pipeline (1) and the following executing command:

The authenticity of the sequenced ancient DNA was estimated by measuring the post-mortem damage rate at the read termini — a classical signature of ancient DNA — and the length of DNA fragments (2).

Genetic sex determination was assigned using three different methods. Firstly, genetic sex was determined calculating X- and Y-ratios (number of reads mapping to each of the sex chromosomes relative to the autosomes) using SexDetERRmine (https://github.com/nf-core/modules/tree/master/modules/nf-core/sexdeterrmine). An X-ratio of 1.0 is considered indicative of the presence of two X chromosomes and 0.5 is considered indicative of a single copy of the X chromosome. A Y-ratio of 0.5 indicates the presence of a single copy of the Y chromosome. Secondly, Rx ratios (3) (ratio of the number of reads mapping to the X chromosome to the number of reads to autosomes, all normalized against the overall number of reads to the reference genome) were calculated. If a Rx ratio falls below 0.6 indicates a male sex. Lastly, the X chromosome read dosage (Mx) (4) was calculated using counts of reads mapping to the X chromosome and autosomes and under the assumption that the number of sequenced reads should reflect the chromosome copy numbers and chromosome lengths, two binomial models are constructed and a likelihood ratio test is then used to distinguish between the male and female models. An Mx close to 0.5 or 1 means that the individual is assigned as male or female, respectively. The obtained raw sequencing reads for the enriched libraries were processed as previously described and mapped against the mitochondrial revised Cambridge Reference Sequence (rCRS) (5).

**References**

1. Fellows Yates J, James A, Lamnidis T, Borry M, Andrades Valtueña A, Fagernas Z, et al. Reproducible, Portable, and Efficient Ancient Genome Reconstruction with Nfcore/Eager. PeerJ. 2021;9:1–25. https://doi.org/10.7717/peerj.10947.

2. Neukamm J, Peltzer A, Nieselt K. DamageProfiler: Fast Damage Pattern Calculation for Ancient DNA. Bioinformatics. 2021;37(20):3652–3. https://doi.org/10.1093/bioinformatics/btab190.

3. Mittnik A, Wang C-C, Svoboda J, Krause J. A molecular approach to the sexing of the triple burial at the Upper Paleolithic Site of Dolní Věstonice. PLoS One. 2016;11(10): e0163019. https://doi.org/10.1371/journal.pone.0163019

4. Gower G, Fenderson LE, Salis AT, Helgen KM, van Loenen AL, Heiniger H, et al. Widespread male sex bias in mammal fossil and museum collections. PNAS. 2019;116(38):19019–24. https://doi.org/10.1073/pnas.1903275116

5. Andrews RM, Kubacka I, Chinnery PF, Lightowlers RN, Turnbull DM, Howell N. Reanalysis and revision of the Cambridge reference sequence for human mitochondrial DNA. Nat Genet. 1999;23(147). https://doi.org/10.1038/13779
