# Supplementary material for: Shrouded in history: Unveiling the ways of life of an early Muslim population in Santarém, Portugal (8th– 10th century AD)
Source: PLoS One. 2024 Mar 6;19(3):e0299958. doi: 10.1371/journal.pone.0299958 (PMC10917335; doi:10.1371/journal.pone.0299958)
Supplement: S2 Text — (DOCX) [file pone.0299958.s003.docx]

**Supporting Information (S3 Text)**

**S3.) Bone apatite preservation**

Changes in the mineral bioapatite of bones can occur in the form of recrystallisation, dissolution, crystal growth, secondary mineral phase precipitation and ionic substitution in calcium and phosphate (1–3). Changes in crystal size within the bone apatite, measured with the infrared splitting factor (IRSF) and carbonate content ratio (C/P), are useful indicators of diagenetic alteration. In this study, attenuated total reflection FTIR (ATR-FTIR) was used to assess these factors. Approximately 15mg of raw bone powder was collected from a selection of individuals with a DREMEL® and ground with an agate mortar and pestle to achieve a homogenous consistency. A Bruker Alpha spectrometer with a single-reflection diamond ATR module (120 scans with a spectral resolution of 4 cm^1^, from 4000 to 375 cm^1^) was used to acquire infrared spectra. The ATR technique was used to measure bone diagenesis because it produces accurate results but requires much less sample preparation than transmission FTIR (4). OPUS/Mentor 6.5 software was used to record and analyse the spectra and to correct and normalise the baseline. The Infrared Splitting Factor (IRSF), relative carbonate content (C/P) and relative collagen content (Am/P) were calculated using the peak heights at wavenumbers 565, 603, 1030, 1035, 1415 and 1640 cm^1^ and mean crystal length was calculated from the IRSF (5). An example of the obtained spectra is provided in S3 Fig 1 and a summary of results is in S3 Table 1.

**S3 Fig 1.) ATR-FTIR Spectra (HS-2271)**

**S3 Table 1.) ATR-FTIR analysis of bone apatite quality results**

| **Sample** | **IRSF** | **C/P** | **Am/P** | **Mean Crystal Length (nm)** |
| --- | --- | --- | --- | --- |
| 1647-F | 3.84 | 0.20 | 0.11 | 62.88 |
| HS-2273 | 4.26 | 0.14 | 0.04 | 71.63 |
| HS-2271 | 3.7 | 0.19 | 0.05 | 59.96 |
| 1092-F | 3.9 | 0.17 | 0.06 | 64.13 |
| HS-2079 | 3.9 | 0.16 | 0.05 | 64.13 |
| HS-2215 | 4 | 0.17 | 0.06 | 66.21 |
| HS-867 | 3.8 | 0.23 | 0.06 | 62.04 |
| 1976-F | 4.19 | 0.13 | 0.03 | 70.17 |
| 1782-F | 3.86 | 0.16 | 0.07 | 63.29 |
| HS-2181 | 3.67 | 0.21 | 0.14 | 59.33 |
| Modern Bone Values | 2.5- 3.25  (4) | 0.23- 0.34  (4) | 0.18-0.42  (5) |  |

The IRSF values, although higher than modern bone, are still 5> and thus do not indicate large crystals or a regularly organized lattice structure in the apatite (4), while C/P values below the modern bone range value of 0.23 indicate some carbonate loss but not the formation of secondary calcite within the bone microstructure (2). The decrease in C/P coincides with an increasing IRSF (S3 Fig 2a), but overall these bones do not appear to be significantly altered by diagenetic processes. When the IRSF, C/P and Am/P are compared to the *δ*^13^C_ap_ values (S3 Fig 2b), there is no apparent correlation between the diagenetic proxies and carbon isotope values, meaning that the *δ*^13^C_ap_ values are more likely to reflect the in vivo carbon in the apatite with little diagenetic influence.

**S3 Fig 2a.) FTIR bone preservation quality indicators IRSF vs C/P**

**S3 Fig 2b.) FTIR bone preservation quality indicators compared to *δ*^13^C_ap_**

**References**

1. Garvie-Lok SJ, Varney TL, Katzenberg MA. Preparation of bone carbonate for stable isotope analysis: the effects of treatment time and acid concentration. J Archaeol Sci. 2004;31(6):763–76. https://doi.org/10.1016/j.jas.2003.10.014

2. Sasso GD, Asscher Y, Angelini I, Nodari L, Artioli G. OPEN A universal curve of apatite crystallinity for the assessment of bone integrity and preservation. Sci Rep. 2018;8:1–13. https://doi.org/10.1038/s41598-018-30642-z

3. Yoder CJ, Bartelink EJ. Effects of different sample preparation methods on stable carbon and oxygen isotope values of bone apatite: a comparison of two treatment protocols. Archaeometry. 2010;52(1):115–30. https://doi.org/10.1111/j.1475-4754.2009.00473.x

4. Beasley MM, Bartelink EJ, Taylor L, Miller RM. Comparison of transmission FTIR, ATR, and DRIFT spectra: Implications for assessment of bone bioapatite diagenesis. J Archaeol Sci. 2014;46:16–22. https://doi.org/10.1016/j.jas.2014.03.008

5. Kontopoulos I, Presslee S, Penkman K, Collins MJ. Preparation of bone powder for FTIR-ATR analysis: The particle size effect. Vib Spectrosc. 2018;99:167–77. https://doi.org/10.1016/j.vibspec.2018.09.004
