# Supplementary material for: Shrouded in history: Unveiling the ways of life of an early Muslim population in Santarém, Portugal (8th– 10th century AD)
Source: PLoS One. 2024 Mar 6;19(3):e0299958. doi: 10.1371/journal.pone.0299958 (PMC10917335; doi:10.1371/journal.pone.0299958)
Supplement: S3 Text — (DOCX) [file pone.0299958.s004.docx]

**Supporting Information (S4 Text)**

**S4.) Statistical comparison of results**

The statistical comparison of isotope results, to assess groups within the population, was conducted using the online statistics calculator “Statistics Kingdom, 2017”(1). Non-parametric Kruskal-Wallis tests (when there were three groups i.e. Grave Orientation) and Mann-Whitney *u* tests (when there were two groups i.e. Sex) were used for non-normally distributed data to assess the mean ranks between the groups, whereby the shape of distribution within each group is assumed to be equal. In the cases where a significant difference between groups was found, a Post-Hoc Dunn’s test with a Bonferroni correction was used to identify which pairs were significantly different. A Levene’s test was used to assess if there was a statistically significant difference of variability between the groups.

**S4 Table 1.) Statistical non-parametric tests comparing groups by grave orientation and sex**

|  | ***δ*^13^C_col_** | ***δ*^15^N** | ***δ*^13^C_en_** | ***δ*^34^S** | ***δ*^18^O_DW_** | **^87^Sr/^86^Sr** |
| --- | --- | --- | --- | --- | --- | --- |
| **Grave Orientation** |  |  |  |  |  |  |
| Kruskal-Wallis H test | 6.365 | 0.053 | 10.795 | 1.149 | 2.094 | 2.727 |
| *p* value | 0.0415  (S) | 0.974  (NS) | 0.0045  (S) | 0.563  (NS) | 0.350  (NS) | 0.256  (NS) |
| Dunn’s test with Bonferroni correction | 0.017  Grp1-Grp2 |  | 0.017  Grp1-Grp3; Grp2-Grp3 |  |  |  |
|  |  |  |  |  |  |  |
| Levene’s test  (F) | df_(2,42)_  0.78 | df_(2,42)_  0.39 | df_(2,22)_  2.371 | df_(2,39)_  2.57 | df_(2,28)_  2.022 | df_(2,14)_  0.63 |
| *p* value | 0.465  (NS) | 0.678  (NS) | 0.117  (NS) | 0.09  (NS) | 0.1513  (NS) | 0.544 (NS) |
| **Sex** |  |  |  |  |  |  |
| Mann-Whitney *U* test (Z) | 0.215 | -0.014 | -0.436 | 1.441 | 1.657 | 1.764 |
| *p* value | 0.83  (NS) | 0.989  (NS) | 0.663  (NS) | 0.15  (NS) | 0.098  (NS) | 0.078  (NS) |
| Levene’s test  (F) | df_(1,37)_  0.024 | df_(1,37)_  0.926 | df_(1,29)_  1.526 | df_(1,29)_  1.83 | df_(1,29)_  3.936 | df_(1,20)_  0.083 |
| *p* value | 0.878  (NS) | 0.342  (NS) | 0.227  (NS) | 0.187  (NS) | 0.057  (NS) | 0.777  (NS) |

1. Statistics Kingdom [Website]. Statistics Calculator. 2017 [Accessed 18 Apr 2023]. http://www.statskingdom.com
